# Long noncoding RNA LINC01578 drives colon cancer metastasis through a positive feedback loop with the NF‐κB/YY1 axis

**DOI:** 10.1002/1878-0261.12819

**Published:** 2020-10-25

**Authors:** Jia Liu, Yang Zhan, Jiefu Wang, Junfeng Wang, Jiansheng Guo, Dalu Kong

**Affiliations:** ^1^ Department of Colorectal Cancer Key Laboratory of Cancer Prevention and Therapy of Tianjin Tianjin's Clinical Research Center for Cancer National Clinical Research Center for Cancer Tianjin Medical University Cancer Institute and Hospital Tianjin China

**Keywords:** colon cancer, feedback loop, long noncoding RNA, metastasis, NF‐κB signaling, YY1

## Abstract

Metastasis accounts for poor prognosis of cancers and related deaths. Accumulating evidence has shown that long noncoding RNAs (lncRNAs) play critical roles in several types of cancer. However, which lncRNAs contribute to metastasis of colon cancer is still largely unknown. In this study, we found that lncRNA LINC01578 was correlated with metastasis and poor prognosis of colon cancer. LINC01578 was upregulated in colon cancer, associated with metastasis, advanced clinical stages, poor overall survival, disease‐specific survival, and disease‐free survival. Gain‐of‐function and loss‐of‐function assays revealed that LINC01578 enhanced colon cancer cell viability and mobility *in vitro* and colon cancer liver metastasis *in vivo*. Mechanistically, nuclear factor kappa B (NF‐κB) and Yin Yang 1 (YY1) directly bound to the *LINC01578* promoter, enhanced its activity, and activated *LINC01578* expression. LINC01578 was shown to be a chromatin‐bound lncRNA, which directly bound *NFKBIB* promoter. Furthermore, LINC01578 interacted with and recruited EZH2 to *NFKBIB* promoter and further repressed *NFKBIB* expression, thereby activating NF‐κB signaling. Through activation of NF‐κB, LINC01578 further upregulated YY1 expression. Through activation of the NF‐κB/YY1 axis, LINC01578 in turn enhanced its own promoter activity, suggesting that LINC01578 and NF‐κB/YY1 formed a positive feedback loop. Blocking NF‐κB signaling abolished the oncogenic roles of LINC01578 in colon cancer. Furthermore, the expression levels of LINC01578, NFKBIB, and YY1 were correlated in clinical tissues. Collectively, this study demonstrated that LINC01578 promoted colon cancer metastasis via forming a positive feedback loop with NF‐κB/YY1 and suggested that LINC01578 represents a potential prognostic biomarker and therapeutic target for colon cancer metastasis.

## Graphical abstract

LINC01578 was upregulated and correlated with metastasis and prognosis in colon cancer. Nuclear factor kappa B (NF‐κB) and Yin Yang 1 (YY1) bound to *LINC01578* promoter to activate *LINC01578* expression. LINC01578 interacted with and recruited EZH2 to *NFKBIB* promoter to repress *NFKBIB* expression, thereby activating NF‐κB signaling. LINC01578 upregulated YY1 via activating NF‐κB. LINC01578 enhanced colon cancer liver metastasis through forming a positive feedback loop with NF‐κB/YY1.

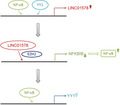

**Abbreviations**
ChIP: chromatin immunoprecipitation
ChIRP: chromatin isolation by RNA purification
COAD: colon adenocarcinoma
CPAT: Coding‐Potential Assessment Tool
CPC: coding potential calculator
DFS: disease‐free survival
DSS: disease‐specific survival
EdU: 5‐ethynyl‐2'‐deoxyuridine
H&E: hematoxylin and eosin
HR: hazard ratio
IHC: immunohistochemistry
IKK: IκB kinase
IκB: inhibitory κB
lncRNAs: long noncoding RNAs
NC: negative control
NCBI: National Center for Biotechnology Information
NF‐κB: nuclear factor kappa B
qRT‐PCR: quantitative real‐time polymerase chain reaction
RIP: RNA immunoprecipitation
RPISeq: RNA‐Protein Interaction Prediction
TCGA: The Cancer Genome Atlas
TNF: tumor necrosis factor
TUNEL: TdT‐mediated dUTP Nick‐End Labeling
YY1: Yin Yang 1

## 1 Introduction

Colon cancer is the fourth most common malignancies and the fifth leading cause of cancer‐related death worldwide [1]. A total of 1 096 601 newly diagnosed colon cancer cases and 551 269 deaths were estimated to occur in 2018 globally [1]. Colon cancer is the fourth most common malignancies and the second leading cause of cancer‐related death in the United States with 104 610 newly diagnosed colon cancer cases and 53 200 deaths estimated to occur in 2020 [2]. Therefore, colon cancer is still an important healthy challenge for humans. Due to the ineffectiveness of early diagnosis, ~ 15–25% of CRC patients are diagnosed with metastasis [3]. Metastasis is the leading cause of cancer‐related death [4]. Although primary colon cancer could be effectively resected, currently therapeutic methods for colon cancer metastasis are still far from satisfactory [5]. Therefore, better understanding of the molecular mechanisms underlying colon cancer metastasis is imperative for the development of more effective therapeutic strategies against colon cancer metastasis.

Metastasis is a complex process, which involves many genetic and epigenetic aberrations [6, 7]. Several molecules and signaling pathways are involved in colon cancer metastatic processes, such as p53, APC, transforming growth factor‐β, nuclear factor kappa B (NF‐κB), and epithelial‐to‐mesenchymal transition [8, 9, 10, 11]. As a crucial transcription factor involved in inflammation and carcinogenesis, NF‐κB contains five main subunits p65, p50/p105, p52/p100, RelB, and c‐Rel. Upon stimulation, activated IκB kinase (IKK) phosphorylates inhibitory κB (IκB) and further induces IκB ubiquitination and degradation. In normal condition, IκB arrests NF‐κB in the cytoplasm. Upon activation, the arrested NF‐κB is released and translocated into the nucleus to modulate target expression [12]. Besides protein‐encoding genes, human genome also encodes more noncoding RNAs. Recent genomic and transcriptomic sequencings have found that < 2% of human genome encode proteins, while > 80% of human genome encode transcripts [13]. Therefore, noncoding RNAs represent the majority of human transcripts [14]. Long noncoding RNAs (lncRNAs) is a class of epigenetically regulatory noncoding RNAs with > 200 nucleotides in length and no protein‐coding ability [15, 16]. Accumulating evidence has identified that a variety of lncRNAs are involved in many pathophysiological processes [17, 18, 19, 20, 21]. Many aberrantly expressed lncRNAs have been found in diseases using high‐throughput RNA sequencing or microarray tests [22, 23]. Furthermore, several lncRNAs are experimentally demonstrated to exert critical roles in diseases progression and regulate almost any of the cancer hallmarks [24, 25, 26]. LncRNAs LDLRAD4‐AS1, Linc00152, SNHG15, CCAL, and lncRNA‐APC1 were reported to have oncogenic or tumor suppressive roles in colon cancer [27, 28, 29, 30, 31]. However, the roles of lncRNAs in colon cancer metastasis are still largely unclear.

Given that metastasis is the leading cause of colon cancer‐related death, we hypothesized that the lncRNAs involved in colon cancer metastasis should also be associated with colon cancer patients' survival. Therefore, we first searched the lncRNAs associated with colon cancer patients' survival using The Cancer Genome Atlas (TCGA) colon adenocarcinoma (COAD) data. Through the analysis of the TCGA COAD data, we found lncRNA LINC01578 was associated with poor overall survival of colon cancer patients. *LINC01578*, named as *CHASERR* in a previous report, is a conserved lncRNA locating at chromatin 15q26.1 and upstream of *CHD2* [32]. Mouse Linc01578 is reported to be essential for cell viability in neurological disease [32]. Here, we further studied the expression pattern, biological roles, and molecular mechanisms of LINC01578 in human colon cancer metastasis.

## 2 Materials and methods

### 2.1 Clinical specimens

Seventy pairs of colon cancer and matched normal tissues were acquired at Tianjin Medical University Cancer Institute and Hospital from colon cancer patients who received surgical resection. The inclusion criteria included (a) histopathologically confirmed colon cancer; and (b) curative surgical resection. The exclusion criteria included (a) presurgical chemotherapy or radiotherapy; and (b) history of other cancers. The clinicopathological features of these 70 colon cancer patients are presented in Table 1. Among these 70 colon cancer patients, 10 have liver metastases and the liver metastatic lesions were resected and collected at Tianjin Medical University Cancer Institute and Hospital. All of the patients were followed up on a regular basis and lasted for a median of 57 months. Disease‐specific survival (DSS) time was defined as the date of surgery to the date of colon cancer‐caused death or the date of the last follow‐up. Disease‐free survival (DFS) time was defined as the date of surgery to the date of colon cancer recurrence or the date of the last follow‐up. This study conformed to the standards set by the Declaration of Helsinki, and was reviewed and approved by the Ethics Committee of Tianjin Medical University Cancer Institute and Hospital. Written informed consents were acquired from all participants.

**Table 1:** Association between LINC01578 and clinicopathological characteristics of colon cancer. *P* value was calculated by chi‐square test. MMR, mismatch repair; MSS, microsatellite stability; pM, pathological staging of metastasis; pN, pathological staging of lymph node; pT, pathological staging of tumor.

Variables	LINC01578 expression		*P* value
	Low	High	
Gender			
Male	19	18	0.811
Female	16	17	
Age			
< 60	18	13	0.229
≥ 60	17	22	
Grade			
Well/moderate	30	27	0.356
Poor	5	8	
MSI status/MMR status			
MSS/MMR‐proficient	22	25	0.445
MSI/MMR‐deficient	13	10	
pT stage			
T1 + 2	9	5	0.232
T3 + 4	26	30	
pN stage			
N0	24	14	0.016
N1 + 2	11	21	
pM stage			
M0	33	27	0.040
M1	2	8	
Clinical stage			
I + II	24	12	0.004
III + IV	11	23	

### 2.2 Cell culture and treatment

Colon mucosa epithelial cell line NCM460, and colon cancer cell lines SW480, HT‐29, LoVo, DLD‐1, HCT116, and Caco‐2 were purchased from American Type Culture Collection (ATCC, Manassas, VA, USA). NCM460, SW480, HT‐29 and HCT116, LoVo, DLD‐1, and Caco‐2 cells were maintained in Dulbecco's modified Eagle's medium (Gibco, Carlsbad, CA, USA), L‐15 Medium (Gibco), McCoy's 5a Medium (Gibco), F­12K Medium (Gibco), RPMI­1640 Medium (Gibco), and Eagle's Minimum Essential Medium (Gibco), respectively. All media were supplied with 10% FBS (Gibco) to make the complete growth medium. All cells were cultured at 37 °C in a 5% CO_2_ incubator. Where indicated, cells were treated with 10 ng·mL^−1^ tumor necrosis factor (TNF)‐α (R&D Systems, Minneapolis, MN, USA), 5 µm JSH‐23 (Selleck), or 5 µm Bay 11‐7085 (Selleck, Houston, TX, USA).

### 2.3 Plasmids, siRNAs, and lentivirus construction

p65 and Yin Yang 1 (YY1) overexpression plasmids were obtained from (GeneCopoeia, Rockville, MD, USA). ON‐TARGET plus Human p65 and YY1 siRNA SMART Pool was obtained from (Dharmacon, Cambridge, UK) (Cat. L‐003533‐00‐0005 and L‐011796‐00‐0005). LINC01578 full‐length sequences were PCR‐amplified with the primers 5′‐CGGGATCCATGGCCGGAGAGGCAGCA‐3′ (forward) and 5′‐GGAATTCTGGCAGGGTGCAATTCATTTA‐3′ (reverse). Next, the PCR products were inserted into the BamH I and EcoR I sites of pcDNA3.1(+) vector (Invitrogen, Carlsbad, CA, USA) and pSPT19 vector (Roche, Basel, Switzerland) to construct LINC01578 overexpression plasmid pcDNA3.1‐LINC01578 and *in vitro* transcription plasmid pSPT19‐LINC01578, respectively. *NFKBIB* promoter binding region‐mutated LINC01578 (LINC01578‐mut) overexpression plasmid pcDNA3.1‐LINC01578‐mut was synthesized by GenScript (Nanjing, Jiangsu, China). Two pairs of cDNA oligonucleotides targeting LINC01578 were synthesized by GenePharma (Shanghai, China). After annealing, double‐strand oligos were cloned into the shRNA lentivirus expressing vector pLV3/H1/GFP&Puro (GenePharma). The constructed vectors were cotransfected with pGag/Pol, pRev, and pVSV‐G (GenePharma) into HEK‐293FT cells to produce shRNA lentivirus targeting LINC01578, termed as LV‐shLINC‐1 and LV‐shLINC‐2. Scrambled shRNA lentivirus served as negative control (LV‐shNC). The shRNAs sequences were as follows: for LV‐shLINC‐1, 5′‐GATCCGCTGTTGGTTCTGAACAAATATTCAAGAGATATTTGTTCAGAACCAACAGCTTTTTTG‐3′ (forward) and 5′‐AATTCAAAAAAGCTGTTGGTTCTGAACAAATATCTCTTGAATATTTGTTCAGAACCAACAGCG‐3′ (reverse); for LV‐shLINC‐2, 5′‐GATCCGAAGTAGACATTGGTGGAAACTTCAAGAGAGTTTCCACCAATGTCTACTTCTTTTTTG‐3′ (forward) and 5′‐AATTCAAAAAAGAAGTAGACATTGGTGGAAACTCTCTTGAAGTTTCCACCAATGTCTACTTCG‐3′ (reverse); and for LV‐shNC, 5′‐GATCCGTTCTCCGAACGTGTCACGTTTCAAGAGAACGTGACACGTTCGGAGAACTTTTTTG‐3′ (forward) and 5′‐AATTCAAAAAAGTTCTCCGAACGTGTCACGTTCTCTTGAAACGTGACACGTTCGGAGAACG‐3′ (reverse). *LINC01578* promoter sequences were PCR‐amplified from genomic DNA using the primers 5′‐GGGGTACCATCGCATCTCAACTCGGATAC‐3′ (forward) and 5′‐CCCAAGCTTCGGAACGCCTGACAGCTC‐3′ (reverse). Next, the PCR products were inserted into the Kpn I and Hind III sites of pGL3‐basic vector (Promega, Madison, WI, USA) to construct *LINC01578* promoter luciferase reporter plasmid pGL3‐LINC01578. NF‐κB transcriptional activity luciferase reporter plasmid pNFκB‐luc was obtained from Beyotime (Shanghai, China). The transfection of plasmids and siRNAs was conducted with Lipofectamine 3000 (Invitrogen) following the manufacturer's manual.

### 2.4 Generation of stable cell lines

To construct colon cancer cells with LINC01578 stable overexpression, LINC01578 overexpression plasmid pcDNA3.1‐LINC01578 was transfected into HCT116 and DLD‐1 cells. Forty‐eight hours after transfection, HCT116 and DLD‐1 cells were treated with neomycin (1000 µg·mL^−1^) for 4 weeks to select LINC01578‐overexpressed cells. To construct LINC01578 stably silenced colon cancer cells, LoVo and HT‐29 cells were infected with LINC01578 shRNAs lentivirus. Ninety‐six hours after infection, the cells were treated with puromycin (2 µg·mL^−1^) for 4 weeks to select LINC01578‐depleted cells. The overexpression and depletion efficiencies were verified using qRT‐PCR as below described.

### 2.5 Quantitative real‐time polymerase chain reaction (qRT‐PCR)

Total RNA was isolated from indicted cells or tissues with TRIzol Reagent (Invitrogen). The RNA was converted into first‐strand cDNA using the PrimeScript™ II 1st Strand cDNA Synthesis Kit (TaKaRa, Shiga, Japan). qRT‐PCR was conducted using TB Green® Premix Ex Taq™ II (Tli RNaseH Plus), Bulk (TaKaRa) on StepOnePlus Real‐Time PCR System (Applied Biosystems, Waltham, MA, USA). GAPDH served as an endogenous control. The primer sequences were as follows: for LINC01578 (NR_037600), 5′‐GCTCGGACCCGGTGACTTA‐3′ (forward) and 5′‐CTCTTCAAGCTGACTGGGTG‐3′ (reverse); for LINC01578 (NR_037601), 5′‐TTTGGGCGACCAGGAGGT‐3′ (forward) and 5′‐TGCAGCCGTTTCAAAATCG‐3′ (reverse); for LINC01578 (NR_037602), 5′‐AGAGCCCCTTTCTGTCCT‐3′ (forward) and 5′‐ATGTTTCCACCAATGTCTAC‐3′ (reverse); for IκBα, 5′‐CACCAACCAGCCAGAAAT‐3′ (forward) and 5′‐CACCCAAGGACACCAAAA‐3′ (reverse); for IκBβ, 5′‐ACCGTACTCCCGACACCAA‐3′ (forward) and 5′‐CGGACCATCTCCACATCTTT‐3′ (reverse); for YY1, 5′‐TCTCAGATCCCAAACAACT‐3′ (forward) and 5′‐AAAGGGCTTCTCTCCAGT‐3′ (reverse); for HEIH, 5′‐CCTCTTGTGCCCCTTTCTT‐3′ (forward) and 5′‐ATGGCTTCTCGCATCCTAT‐3′ (reverse); and for GAPDH, 5′‐GTCGGAGTCAACGGATTTG‐3′ (forward) and 5′‐TGGGTGGAATCATATTGGAA‐3′ (reverse). Relative quantification was calculated using the comparative C*_t_* method.

### 2.6 Western blot

Western blot was conducted as previously described [33]. Briefly, total protein was isolated from indicted cells using RIPA Lysis Buffer (Beyotime) supplied with protease inhibitor. Nuclear protein was isolated from indicated cells with the Nuclear Extraction Kit (ab113474; Abcam, Cambridge, UK). The extracted proteins were loaded onto SDS/PAGE for separation, followed by being transferred onto polyvinylidene fluoride membrane. Primary antibodies used were as follows: YY1 (Cat. 712089, 1/500 dilution; Invitrogen); GAPDH (Cat. ab8245, 1/5000 dilution; Abcam); p50 (Cat. #13586, 1/1000 dilution; Cell Signaling Technology, Danvers, MA, USA); p65 (Cat. #8242, 1/1000 dilution; Cell Signaling Technology); histone H3 (Cat. ab1791, 1/1000 dilution; Abcam); IκBβ (Cat. ab109509, 1/1000 dilution; Abcam); and EZH2 (Cat. ab195409, 1/1000 dilution; Abcam). GAPDH and histone H3 were used as endogenous control for total protein and nuclear protein, respectively.

### 2.7 Chromatin immunoprecipitation (ChIP)

ChIP assay was conducted in indicated colon cancer cells with the EZ‐Magna ChIP™ A/G ChIP Kit (Millipore, Billerica, MA, USA) and 5 µg antibodies according to the provided manual. The antibodies used were as follows: YY1 (Cat. 712089; Invitrogen); p50 (Cat. #13586; Cell Signaling Technology); p65 (Cat. #8242; Cell Signaling Technology); H3K27me3 (Cat. 17‐622; Millipore); H3K27ac (Cat. 17‐683; Millipore); and EZH2 (Cat. ab195409; Abcam). The enriched DNA was measured using qPCR with the primers: for *LINC01578* promoter (P1), 5′‐TGTTGTGTGGTGTGTGGGG‐3′ (forward) and 5′‐TGACAGCTCGGGGTGCTG‐3′ (reverse); for *LINC01578* promoter (P2), 5′‐TTGTGCGACCCTGCACTA‐3′ (forward) and 5′‐TTCAACACCACCCCTTCC‐3′ (reverse); for *LINC01578* promoter control (P3), 5′‐AGTATTTTGTGGGTGTGTGTG‐3′ (forward) and 5′‐TTGGCTCAGTGTAGCATCA‐3′ (reverse); for *NFKBIB* promoter, 5′‐TCCTCCCGAATACTCTGA‐3′ (forward) and 5′‐CTGTCGCACCATTCATCT‐3′ (reverse); and for *NFKBIB* promoter NC, 5′‐GGAAGATGAAATTGAGATGAGG‐3′ (forward) and 5′‐AGACAAGGTGAGAAATGGTGA‐3′ (reverse).

### 2.8 Luciferase reporter assay

To measure *LINC01578* promoter activity, *LINC01578* promoter firefly luciferase reporter plasmid pGL3‐LINC01578 was cotransfected with pRL‐TK into indicated colon cancer cells. pRL‐TK, which encodes Renilla luciferase, was used as endogenous control. Forty‐eight hours after transfection, the firefly and Renilla luciferase activities were detected by the Dual‐Luciferase Reporter Assay System (Promega). *LINC01578* promoter activity was calculated as the ratio of firefly luciferase activity to Renilla luciferase activity. To measure NF‐κB transcriptional activity, firefly luciferase reporter plasmid pNFκB‐luc was cotransfected with pRL‐TK into indicated colon cancer cells. Forty‐eight hours after transfection, the firefly and Renilla luciferase activities were detected by the Dual‐Luciferase Reporter Assay System (Promega). NF‐κB transcriptional activity was calculated as the ratio of firefly luciferase activity to Renilla luciferase activity.

### 2.9 NF‐κB activation measurement

The nuclear extracts from indicated cells were used to measure NF‐κB transcription factor DNA binding activities using the NF‐κB p50 Transcription Factor Assay Kit (Cat. ab207217; Abcam) and NF‐κB p65 Transcription Factor Assay Kit (Cat. ab133112; Abcam) following the manufacturer's protocols.

### 2.10 Subcellular fractionation and polyadenylated RNA enrichment

Subcellular fractionation was conducted as previously described [34]. The RNA in different subcellular components was extracted and detected using qRT‐PCR as above described. Polyadenylated RNA was captured and purified using oligo(dT) polystyrene beads (GenElute mRNA Miniprep Kit; Sigma‐Aldrich, St Louis, MO, USA). The enriched polyadenylated RNA was detected using qRT‐PCR as above described.

### 2.11 Chromatin Isolation by RNA Purification (ChIRP)

ChIRP was conducted using the Magna ChIRP™ RNA Interactome Kit (Millipore) according to the provided protocol. The sequences of the antisense DNA probes against LINC01578 were as follows: 1, 5′‐TTAAAAAGCTCCTCCGTTGG‐3′; 2, 5′‐ACTGCAGCCGTTTCAAAATC‐3′; 3, 5′‐CCAACGTCTCAGTCTTCAAA‐3′; 4, 5′‐GTCTACTTCATTGTATGGGG‐3′; 5, 5′‐CATAGTAGACTGCCATCTTG‐3′; 6, 5′‐TTCACTGCATGGAATCCATG‐3′; 7, 5′‐CTCCAAATGTCAAAATGTCC‐3′; 8, 5′‐AGAGGTCATACGCATACAAA‐3′; 9, 5′‐TTACCACTCGGCAACTACAA‐3′; 10, 5′‐ACCATCTAAGAGCACAACTT‐3′; 11, 5′‐TCAGATTCTATTATTACCCA‐3′; and 12, 5′‐ATATGTGTATCACTCATCCT‐3′. The LacZ probes provided in this kit were used as NC. The retrieved DNA was measured using qPCR with the primers 5′‐TCCTCCCGAATACTCTGA‐3′ (forward) and 5′‐CTGTCGCACCATTCATCT‐3′ (reverse) for *NFKBIB* promoter; and 5′‐GGAAGATGAAATTGAGATGAGG‐3′ (forward) and 5′‐AGACAAGGTGAGAAATGGTGA‐3′ (reverse) for *NFKBIB* promoter NC.

### 2.12 RNA pull‐down

RNA pull‐down assays were conducted as previously described [34]. Briefly, LINC01578 and antisense LINC01578 were *in vitro*‐transcribed and biotin‐labeled from pSPT19‐LINC01578 by the Biotin RNA Labeling Mix (Roche) and T7 and Sp6 RNA polymerase (Roche), respectively. After being purified, 3 µg of *in vitro*‐transcribed LINC01578 was incubated with 1 mg of whole‐cell lysate of DLD‐1 cells at 25 °C for 1 h. Next, the complexes were enriched by streptavidin agarose beads (Invitrogen). The proteins present in the pull‐down material were measured using western blot.

### 2.13 RNA immunoprecipitation (RIP)

RIP assay was conducted by the Magna RIP™ RNA‐Binding Protein Immunoprecipitation Kit (Millipore) and primary antibody against EZH2 (Cat. ab195409; Abcam). The enriched RNA was measured using qRT‐PCR.

### 2.14 Cell viability and proliferation assays

Glo cell viability assay was conducted to measure cell viability. 5‐Ethynyl‐2'‐deoxyuridine (EdU) assay was conducted to measure cell proliferation. Glo cell viability and EdU assays were carried out as previously described [35]. Briefly, Glo cell viability assay was conducted with the CellTiter‐Glo® Luminescent Cell Viability Assay (Cat. G7570; Promega). EdU assay was conducted with the EdU Kit (Cat. C00031; RiboBio, Guangzhou, China). The percentage of EdU‐positive cell was calculated using a fluorescence microscope based on at least five random fields.

### 2.15 Cell migration and invasion assays

Cell migration and invasion abilities were detected by transwell migration and invasion assays as previously described [27]. Briefly, 50 000 indicated cells were seeded into the upper chamber of 24‐well transwell insert precoated with (invasion) or without (migration) Matrigel. After culture for 48 h, the cells remaining in the upper chamber were removed. The migrated or invasive cells were fixed, stained, and counted using a microscope based on at least five random fields.

### 2.16 Mice studies

Five‐week‐old male BALB/c athymic nude mice were used for animal studies. For liver metastasis experiments, 3 × 10^6^ indicated cells were injected into the spleen of nude mice. The mice were allowed to grow for another 6 weeks. Next, the livers were resected and liver metastases were detected by hematoxylin and eosin (H&E) staining. The liver metastatic lesions were further used to perform immunohistochemistry (IHC) staining as previously described [36] with primary antibodies against Ki67 (Cat. ab16667; Abcam), p50 (Cat. ab32360; Abcam), or YY1 (Cat. ab109228; Abcam). In addition, the liver metastatic lesions were also used to perform TdT‐mediated dUTP Nick‐End Labeling (TUNEL) staining using the One Step TUNEL Apoptosis Assay Kit (Cat. C1086; Beyotime). Mice studies conformed to the animal care guidelines and were approved by the Ethics Committee of Tianjin Medical University Cancer Institute and Hospital.

### 2.17 Bioinformatics analysis

The correlation between lncRNA expression and prognosis of colon cancer patients from TCGA COAD data was analyzed using the online *in silico* tool GEPIA (http://gepia.cancer‐pku.cn/). The normalized expression data of LINC01578 (probe number: 225952_s_at), NFKBIB (probe number: 214448_x_at), and YY1 (probe number: 200047_s_at) from GSE17538 and GSE37892 were download from Gene Expression Omnibus (GEO) database (https://www.ncbi.nlm.nih.gov/geo/). Coding potential of long RNAs was analyzed using online *in silico* tools including the Coding‐Potential Assessment Tool (CPAT) (http://lilab.research.bcm.edu/cpat/), the coding potential calculator (CPC) (http://cpc.cbi.pku.edu.cn/), and PhyloCSF from UCSC Genome Browser (http://genome.ucsc.edu/). Transcription factor binding sites were predicted using the online *in silico* tool JASPAR (http://jaspar.genereg.net/). The interaction probability between lncRNAs and proteins was predicted using the online *in silico* tool RNA‐Protein Interaction Prediction (RPISeq) (http://pridb.gdcb.iastate.edu/RPISeq/). Interaction probabilities range from 0 to 1. Predictions with probabilities > 0.5 were considered positive [37, 38].

### 2.18 Statistical analysis

All statistical analyses were conducted by the graphpad prism 6.0 Software, (GraphPad, La Jolla, CA, USA). The detailed statistical methods were described in the figure and table legends. *P* < 0.05 was recognized as statistically significant.

## 3 Results

### 3.1 LINC01578 was correlated with metastasis and poor prognosis of colon cancer

**Fig. 1:**
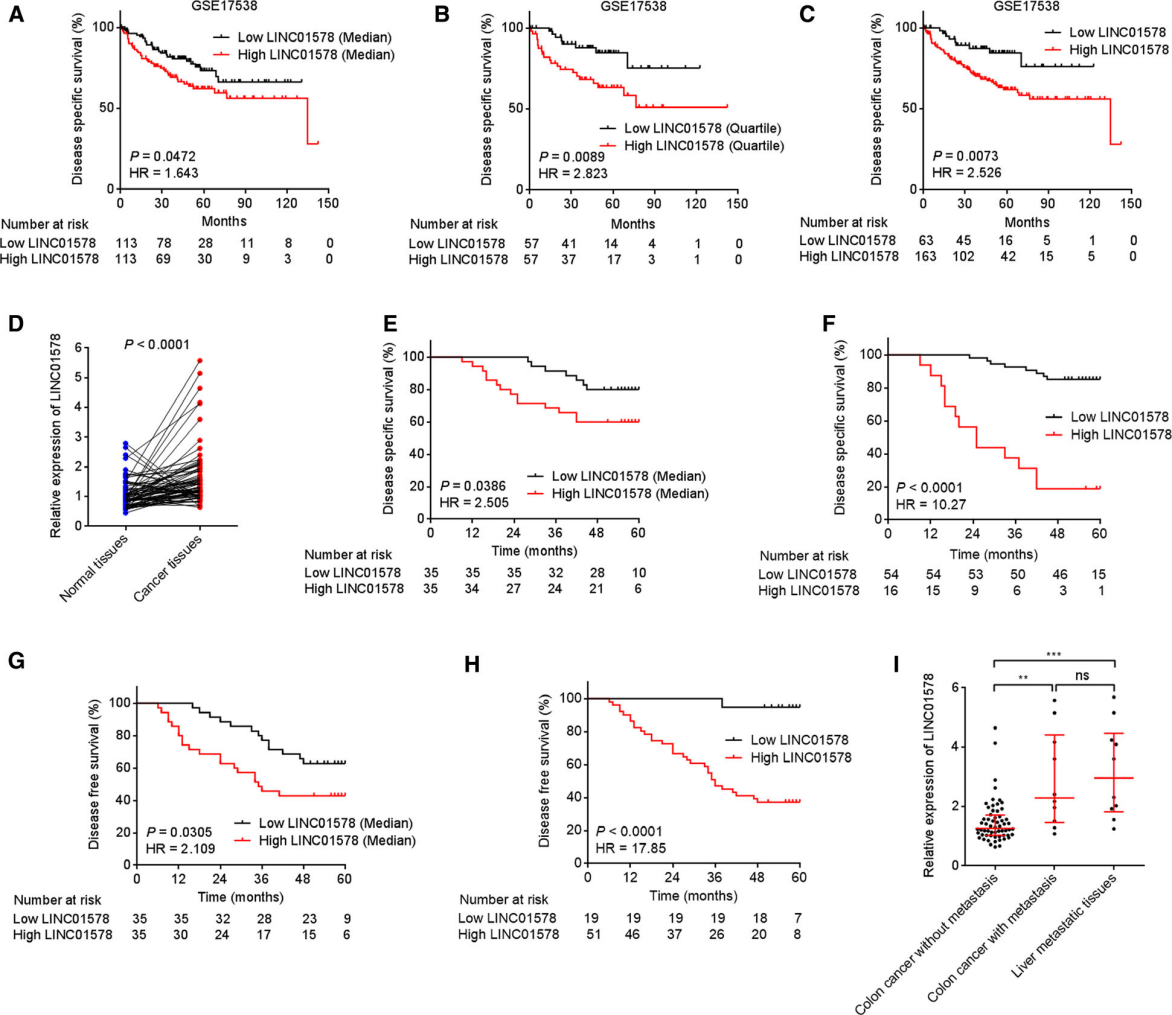
The expression pattern and clinical significance of LINC01578 in colon cancer. (A) Kaplan–Meier survival analysis of the correlation between LINC01578 expression and DSS in GSE17538 dataset. *n* = 226. LINC01578 median level was used as cutoff. *P* = 0.0472, HR = 1.643 by log‐rank test. (B) Kaplan–Meier survival analysis of the correlation between LINC01578 expression and DSS in GSE17538 dataset. *n* = 226. LINC01578 quartile level was used as cutoff. *P* = 0.0089, HR = 2.823 by log‐rank test. (C) Kaplan–Meier survival analysis of the correlation between LINC01578 expression and DSS in GSE17538 dataset. *n* = 226. An optimal cutoff with LINC01578 probe intensity of 9.525 was used. *P* = 0.0073, HR = 2.526 by log‐rank test. (D) LINC01578 expression in 70 pairs of colon cancer tissues and matched normal tissues was measured by qRT‐PCR. Three random selected colon cancer tissues were used as reference. *P* < 0.0001 by Wilcoxon matched‐pairs signed rank test. (E) Kaplan–Meier survival analysis of the correlation between LINC01578 expression and DSS in 70 colon cancer patients. LINC01578 median level was used as cutoff. *P* = 0.0386, HR = 2.505 by log‐rank test. (F) Kaplan–Meier survival analysis of the correlation between LINC01578 expression and DSS in 70 colon cancer patients. An optimal cutoff with LINC01578 relative expression level of 1.998 was used. *P* < 0.0001, HR = 10.27 by log‐rank test. (G) Kaplan–Meier survival analysis of the correlation between LINC01578 expression and DFS in 70 colon cancer patients. LINC01578 median level was used as cutoff. *P* = 0.0305, HR = 2.109 by log‐rank test. (H) Kaplan–Meier survival analysis of the correlation between LINC01578 expression and DFS in 70 colon cancer patients. An optimal cutoff with LINC01578 relative expression level of 1.095 was used. *P* < 0.0001, HR = 17.85 by log‐rank test. (I) LINC01578 expression in 60 colon cancer tissues without metastasis, 10 colon cancer tissues with metastasis, and 10 matched liver metastatic tissues was measured by qRT‐PCR. **P < 0.01, ***P < 0.001, ns, not significant, by the Kruskal–Wallis test followed by Dunn's multiple comparisons test.

To search the lncRNAs associated with outcome of colon cancer patients, we analyzed TCGA COAD data using the GEPIA. The most differentially survival genes are shown in Table S1. Among the top differentially survival genes, we noted lncRNA LINC01578 whose high expression was correlated with remarkably worse overall survival of colon cancer patients with a hazard ratio (HR) value of 2.9 (Fig. S1A). In addition, the GSE17538 dataset that contains 226 colon cancer tissues with prognostic information showed that increased expression of LINC01578 was correlated with worse DSS (Fig. 1A–C). To further elucidate the expression pattern and clinical relevance of LINC01578 in colon cancer, we collected 70 pairs of colon cancer and matched normal tissues. The expression of LINC01578 in these tissues was detected by qRT‐PCR, and the results revealed that LINC01578 was upregulated in colon cancer tissues with respect to normal tissues (Fig. 1D). Correlation analysis between LINC01578 expression and clinicopathological characteristics revealed that high expression of LINC01578 was positively correlated with lymph node metastasis, distant metastasis, and advanced TNM stages, but not correlated with gender, age, grade, and microsatellite instability (MSI) status (Table 1). The Kaplan–Meier analysis revealed that high expression of LINC01578 was correlated with worse DSS (Fig. 1E,F) and DFS (recurrence) (Fig. 1G,H). Furthermore, univariate and multivariate survival analyses revealed that high LINC01578 expression was independently correlated with poor DSS and DFS (Table S2). To elucidate the expression pattern of LINC01578 in colon cancer metastatic processes, we detected the expression of LINC01578 in colon cancer tissues without metastasis, colon cancer tissues with metastasis, and liver metastatic tissues using qRT‐PCR. The results revealed that LINC01578 was remarkably increased in colon cancer tissues with metastasis with respect to colon cancer tissues without metastasis (Fig. 1I). The expression of LINC01578 in liver metastatic tissues was not significantly different from that in matched primary colon cancer tissues (Fig. 1I). GSE37892 dataset that contains 93 colon cancer without metastasis and 37 colon cancer with metastasis presented that LINC01578 was also remarkably increased in colon cancer tissues with metastasis with respect to colon cancer tissues without metastasis (Fig. S1B). In addition, the GSE37892 dataset also showed that LINC01578 was correlated with advanced TNM stages, but not correlated with gender, age, and tumor localization (Fig. S1C‐F). The expression of LINC01578 in colon cancer cell lines was measured by qRT‐PCR, and the results revealed that LINC01578 was increased in all colon cancer cell lines compared with a normal colon mucosa epithelial cell line NCM460 (Fig. S1G). Collectively, these results demonstrated that increased expression of LINC01578 was associated with metastasis and poor prognosis of colon cancer patients.

LINC01578 has three different isoforms annotated by the National Center for Biotechnology Information (NCBI) (https://www.ncbi.nlm.nih.gov/) (Fig. S1H). qRT‐PCR results showed that the isoform NR_037601 was the major transcript in colon cancer cell lines and tissues (Fig. S1I,J). Therefore, in this study we focused on NR_037601. NR_037601 has six exons and 1787 nucleotides in length. Three online *in silico* tools including the CPAT, the CPC, and PhyloCSF all indicate that LINC01578 was a noncoding RNA, with CPAT score of 0.0067, CPC score of −0.9136, and PhyloCSF score of below zero. LINC01578 had a poly (A) tail (Fig. S1K).

### 3.2 LINC01578 was upregulated by NF‐κB and YY1

**Fig. 2:**
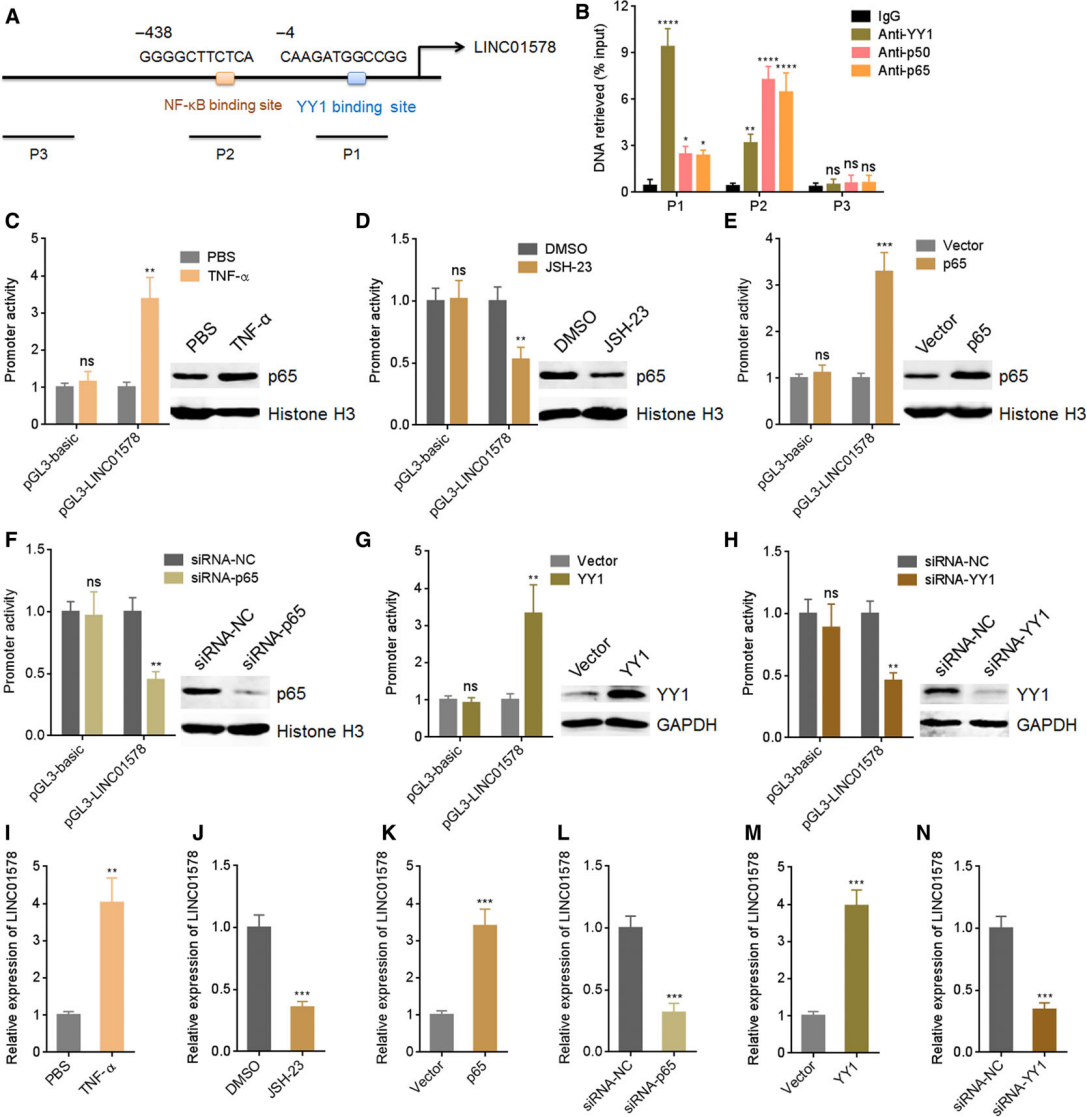
LINC01578 is upregulated by NF‐κB and YY1. (A) The predicted NF‐κB and YY1 binding sites on *LINC01578* promoter. NF‐κB and YY1 bind sites were at −438 and −4 positions relative to the transcription start site, respectively. (B) ChIP assays were performed in DLD‐1 cells to measure the binding of NF‐κB and YY1 on *LINC01578* promoter. A distant region without NF‐κB and YY1 binding sites was used as NC (P3). (C) Luciferase reporter assays for DLD‐1 cells transfected with luciferase reporter plasmids containing *LINC01578* promoter and treated with PBS or 10 ng·mL^−1^ TNF‐α for 24 h. Nuclear p65 levels of DLD‐1 cell treatment with PBS or 10 ng·mL^−1^ TNF‐α for 24 h were detected by western blot. (D) Luciferase reporter assays for DLD‐1 cells transfected with luciferase reporter plasmids containing *LINC01578* promoter and treated with DMSO or 5 µm JSH‐23 for 24 h. Nuclear p65 levels of DLD‐1 cell treatment with DMSO or 5 µm JSH‐23 for 24 h were detected by western blot. (E) Luciferase reporter assays for DLD‐1 cells cotransfected with luciferase reporter plasmids containing *LINC01578* promoter and p65 overexpression vector. p65 overexpression efficiencies were detected by western blot. (F) Luciferase reporter assays for DLD‐1 cells cotransfected with luciferase reporter plasmids containing *LINC01578* promoter and siRNAs against p65. p65 knockdown efficiencies were detected by western blot. (G) Luciferase reporter assays for DLD‐1 cells cotransfected with luciferase reporter plasmids containing *LINC01578* promoter and YY1 overexpression vector. YY1 overexpression efficiencies were detected by western blot. (H) Luciferase reporter assays for DLD‐1 cells cotransfected with luciferase reporter plasmids containing *LINC01578* promoter and siRNAs against YY1. YY1 knockdown efficiencies were detected by western blot. (I) LINC01578 expression in DLD‐1 cells treated with PBS or 10 ng·mL^−1^ TNF‐α for 24 h. (J) LINC01578 expression in DLD‐1 cells treated with DMSO or 5 µm JSH‐23 for 24 h. (K) LINC01578 expression in DLD‐1 cells transfected with p65 overexpression vector. (L) LINC01578 expression in DLD‐1 cells transfected with siRNAs against p65. (M) LINC01578 expression in DLD‐1 cells transfected with YY1 overexpression vector. (N) LINC01578 expression in DLD‐1 cells transfected with siRNAs against YY1. Data are shown as mean ± SD based on three independent experiments. *P < 0.05, **P < 0.01, ***P < 0.001, ****P < 0.0001, ns, not significant, by one‐way ANOVA followed by Dunnett's multiple comparisons test (B) or Student's *t*‐test (C‐N).

To elucidate the factors modulating LINC01578 expression, we searched potential transcription factors binding sites on *LINC01578* promoter using the online *in silico* tool JASPAR (http://jaspar.genereg.net/). Intriguingly, NF‐κB and YY1 binding sites were both predicted on *LINC01578* promoter (Fig. 2A). NF‐κB was revealed to be activated in colon cancer [11]. Moreover, NF‐κB was reported to activate YY1 [39]. Thus, we next studied whether NF‐κB and YY1 modulated LINC01578 expression in colon cancer. ChIP assays demonstrated the specific binding of NF‐κB (p50 and p65) and YY1 on *LINC01578* promoter region but not on a distant control region (Fig. 2B). pGL3 luciferase reporter plasmid assays presented that treatment of DLD‐1 cells with a classical NF‐κB activator TNF‐α significantly increased *LINC01578* promoter activity (Fig. 2C). The activation of NF‐κB was verified by the increased nuclear level of p65 (Fig. 2C). Conversely, treatment with NF‐κB inhibitor JSH‐23 decreased *LINC01578* promoter activity (Fig. 2D). Consistently, overexpression of p65 increased *LINC01578* promoter activity (Fig. 2E). p65 depletion reduced *LINC01578* promoter activity (Fig. 2F). Overexpression of YY1 increased *LINC01578* promoter activity (Fig. 2G). YY1 depletion reduced *LINC01578* promoter activity (Fig. 2H). These results demonstrated that NF‐κB and YY1 activated *LINC01578* promoter activity. Next, the modulation of NF‐κB and YY1 on LINC01578 expression was investigated. Treatment with NF‐κB activator TNF‐α upregulated LINC01578 expression (Fig. 2I). Treatment with NF‐κB inhibitor JSH‐23 decreased LINC01578 expression (Fig. 2J). Overexpression of p65 upregulated LINC01578 expression (Fig. 2K). p65 depletion reduced LINC01578 expression (Fig. 2L). Overexpression of YY1 upregulated LINC01578 expression (Fig. 2M). YY1 depletion reduced LINC01578 expression (Fig. 2N). Furthermore, the effects of NF‐κB and YY1 on LINC01578 were also verified in another colon cancer cell line HT‐29 (Fig. S2). Therefore, these results demonstrated that NF‐κB and YY1 activated LINC01578 expression.

### 3.3 **LINC01578 enhanced colon cancer cell viability and mobility** *in vitro*

**Fig. 3:**
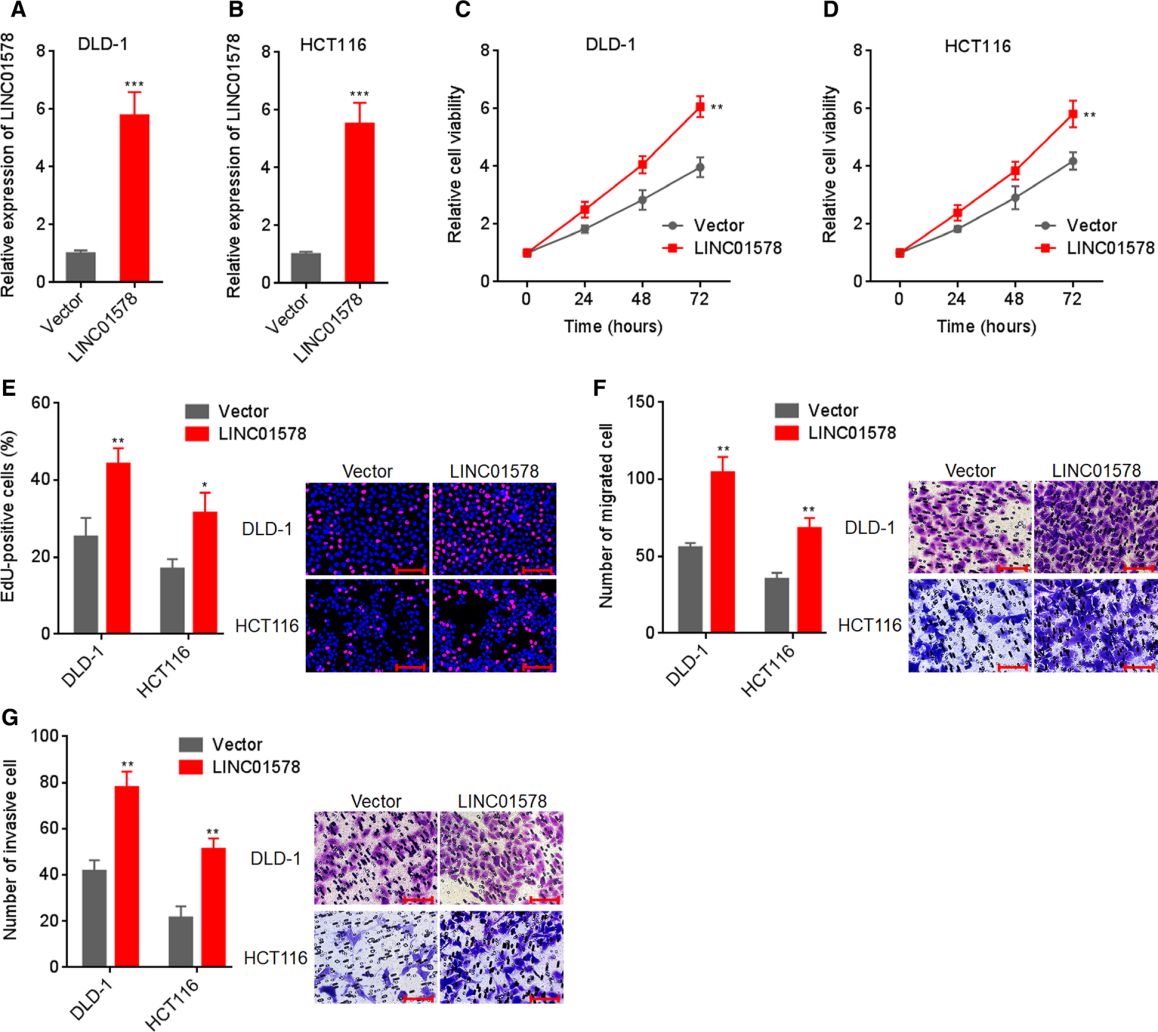
Overexpression of LINC01578 enhanced colon cancer cell viability and mobility. (A) LINC01578 expression in DLD‐1 cells transfected with LINC01578 overexpression vector. (B) LINC01578 expression in HCT116 cells transfected with LINC01578 overexpression vector. (C) Cell viability of LINC01578 overexpressed and control DLD‐1 cells was determined by Glo cell viability assay. (D) Cell viability of LINC01578‐overexpressed and control HCT116 cells was determined by Glo cell viability assay. (E) Cell proliferation of LINC01578‐overexpressed and control DLD‐1 and HCT116 cells was determined by EdU assays. Scale bars, 100 µm. (F) Cell migration of LINC01578‐overexpressed and control DLD‐1 and HCT116 cells was determined by transwell migration assays. Scale bars, 100 µm. (G) Cell invasion of LINC01578‐overexpressed and control DLD‐1 and HCT116 cells was determined by transwell invasion assays. Scale bars, 100 µm. Data are shown as mean ± SD based on three independent experiments. *P < 0.05, **P < 0.01, ***P < 0.001 by Student's *t*‐test.

Considering the correlation between LINC01578 and metastasis and poor prognosis in colon cancer, we further studied whether LINC01578 contributes to colon cancer progression. LINC01578 overexpression plasmid pcDNA3.1‐LINC01578 was transfected into colon cancer cells DLD‐1 and HCT116 to sufficiently increase LINC01578 expression (Fig. 3A,B). Glo cell viability assays showed that cell viabilities of DLD‐1 and HCT116 cells increased significantly after LINC01578 overexpression (Fig. 3C,D). EdU assays showed that LINC01578 overexpression led to significant acceleration in cell proliferation (Fig. 3E). Transwell migration assays presented that cell migration increased remarkably after LINC01578 overexpression (Fig. 3F). Transwell invasion assays presented that cell invasion increased remarkably after LINC01578 overexpression (Fig. 3G). To preclude the possible clonal effect, another LINC01578 overexpressing clone of DLD‐1 cells was constructed (Fig. S3A). Glo cell viability and EdU assays showed that cell viability and proliferation consistently increased after LINC01578 overexpression (Fig. S3B,C). Transwell migration and invasion assays showed that cell migration and invasion consistently increased after LINC01578 overexpression (Fig. S3D,E).

**Fig. 4:**
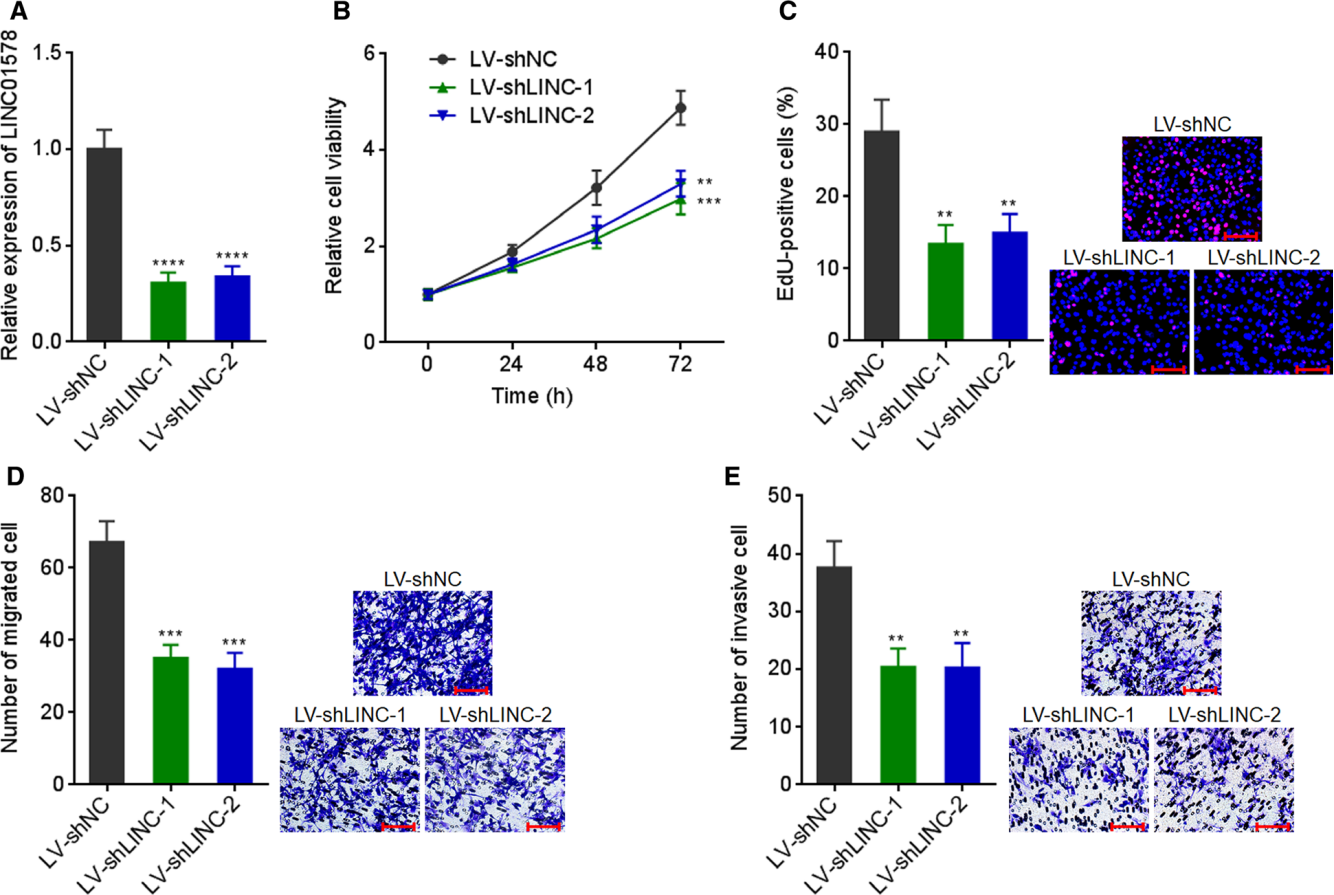
Depletion of LINC01578 repressed colon cancer cell viability and mobility. (A) LINC01578 expression in LoVo cells infected with shRNAs targeted to LINC01578. (B) Cell viability of LINC01578‐depleted and control LoVo cells was determined by Glo cell viability assay. (C) Cell proliferation of LINC01578‐depleted and control LoVo cells was determined by EdU assays. Scale bars, 100 µm. (D) Cell migration of LINC01578‐depleted and control LoVo cells was determined by transwell migration assays. Scale bars, 100 µm. (E) Cell invasion of LINC01578‐depleted and control LoVo cells was determined by transwell invasion assays. Scale bars, 100 µm. Data are shown as mean ± SD based on three independent experiments. **P < 0.01, ***P < 0.001, ****P < 0.0001 by one‐way ANOVA followed by Dunnett's multiple comparisons test.

In addition, two independent short hairpin RNAs (shRNAs) targeting to LINC01578 were infected into colon cancer cells LoVo via lentivirus to sufficiently inhibit LINC01578 expression (Fig. 4A). Glo cell viability assays showed that cell viabilities of LoVo cells reduced significantly after LINC01578 inhibition (Fig. 4B). EdU assays showed that LINC01578 inhibition led to significant repression in cell proliferation (Fig. 4C). Transwell migration assays presented that cell migration reduced remarkably after LINC01578 inhibition (Fig. 4D). Transwell invasion assays presented that cell invasion reduced remarkably after LINC01578 inhibition (Fig. 4E). LINC01578‐silenced another colon cancer cell HT‐29 was constructed via shRNA lentivirus (Fig. S4A). Glo cell viability and EdU assays showed that cell viability and proliferation of HT‐29 cells were reduced after LINC01578 inhibition (Fig. S4B,C). Transwell migration and invasion assays showed that cell migration and invasion of HT‐29 cells were reduced after LINC01578 inhibition (Fig. S4D,E). Collectively, these findings demonstrated that overexpression of LINC01578 enhanced, but depletion of LINC01578 repressed, colon cancer cell viability and mobility *in vitro*.

### 3.4 LINC01578 drove colon cancer metastasis *in vivo*

**Fig. 5:**
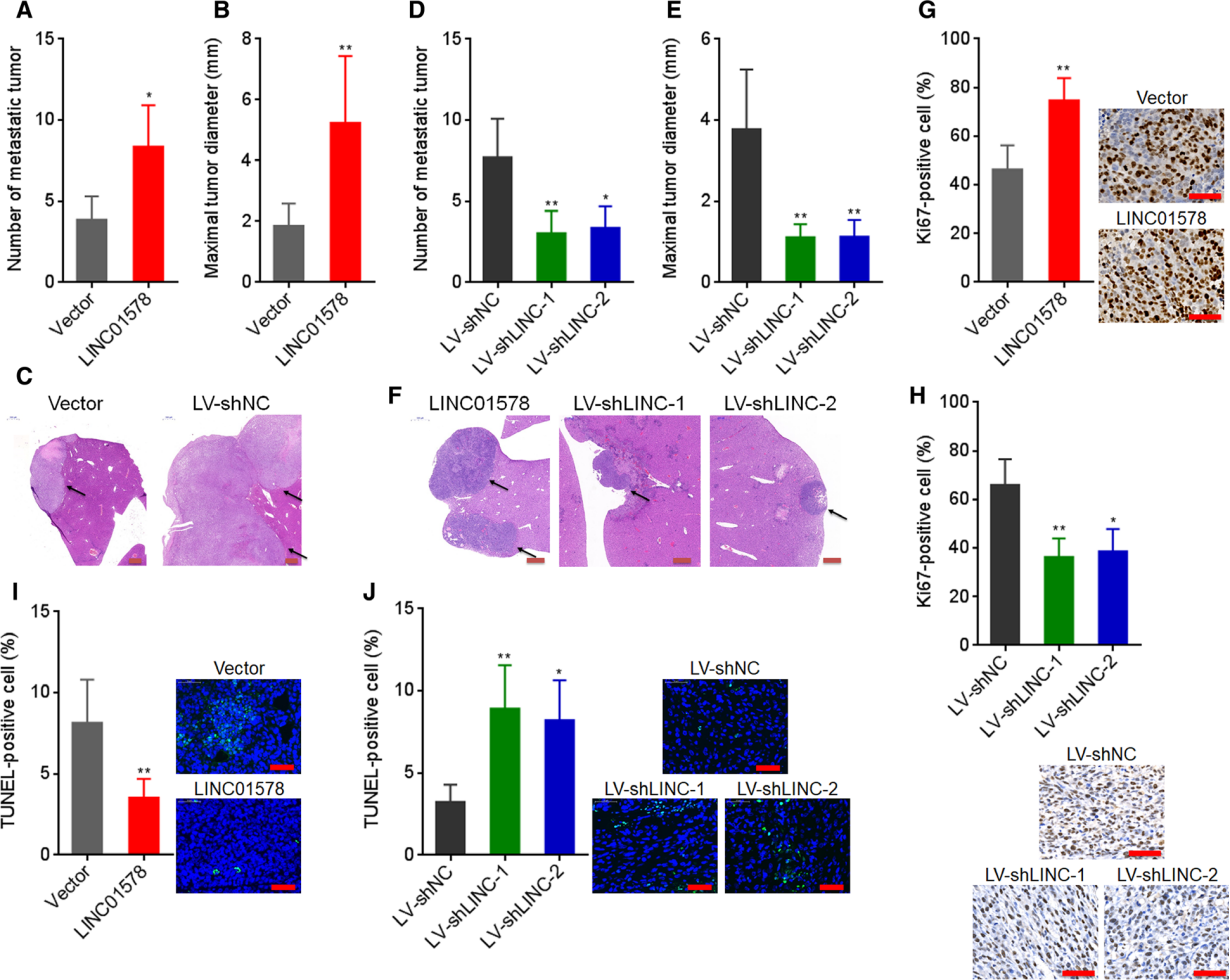
LINC01578 drove colon cancer liver metastasis. (A–C) LINC01578‐overexpressed and control DLD‐1 cells were intrasplenic injected into nude mice. Six weeks after injection, liver metastasis was detected by H&E staining. Statistical analysis of liver metastatic tumor number (A) and maximal liver metastatic tumor diameter (B) are shown. Representative H&E staining of liver metastatic lesions are shown in (C). Scale bars, 500 µm. (D–F) LINC01578‐depleted and control LoVo cells were intrasplenic injected into nude mice. Six weeks after injection, liver metastasis was detected by H&E staining. Statistical analysis of liver metastatic tumor number (D) and maximal liver metastatic tumor diameter (E) is shown. Representative H&E staining of liver metastatic lesions is shown in (F). Scale bars, 500 µm. (G) Ki67 IHC staining of liver metastatic tumors derived from (C). Scale bars, 50 µm. (H) Ki67 IHC staining of liver metastatic tumors derived from (F). Scale bars, 50 µm. (I) TUNEL staining of liver metastatic tumors derived from (C). Scale bars, 50 µm. (J) TUNEL staining of liver metastatic tumors derived from (F). Scale bars, 50 µm. Data are shown as mean ± SD based on *n* = 6 mice in each group. *P < 0.05, **P < 0.01 by the Mann–Whitney test (A,B,G,I) or the Kruskal–Wallis test followed by Dunn's multiple comparisons test (D,E,H,J).

Given the correlation between LINC01578 and metastasis and poor prognosis in colon cancer, we investigated the roles of LINC01578 in colon cancer metastasis *in vivo*. DLD‐1 cells with LINC01578 overexpression or control were injected into spleen of nude mice to construct liver metastasis model. Six weeks after injection, liver metastases were measured by H&E staining. As shown in Fig. 5A–C, DLD‐1 cells formed more and larger liver metastases after LINC01578 overexpression. LINC01578‐depleted and control LoVo cells were also injected into spleen of nude mice. Six weeks after injection, liver metastases were evaluated by H&E staining. As shown in Fig. 5D–F, LoVo cells formed less and smaller liver metastases after LINC01578 depletion. Proliferation marker Ki67 IHC staining presented that the liver metastatic tumors formed by DLD‐1 cells with LINC01578 overexpression had more Ki67‐positive cells with respect to the liver metastatic tumors formed by control DLD‐1 cells (Fig. 5G). Conversely, the liver metastatic tumors formed by LINC01578‐depleted LoVo cells had less Ki67‐positive cells with respect to the liver metastatic tumors formed by control LoVo cells (Fig. 5H). Apoptosis marker TUNEL staining showed that the liver metastatic tumors formed by DLD‐1 cells with LINC01578 overexpression had less TUNEL‐positive cells with respect to the liver metastatic tumors formed by control DLD‐1 cells (Fig. 5I). Conversely, the liver metastatic tumors formed by LINC01578‐depleted LoVo cells had more TUNEL‐positive cells with respect to the liver metastatic tumors formed by control LoVo cells (Fig. 5J). Taken together, these findings demonstrated that LINC01578 drove colon cancer liver metastasis, promoted colon cancer cell proliferation, and repressed colon cancer cell apoptosis *in vivo*.

### 3.5 **LINC01578 activated NF**‐**κB**

**Fig. 6:**
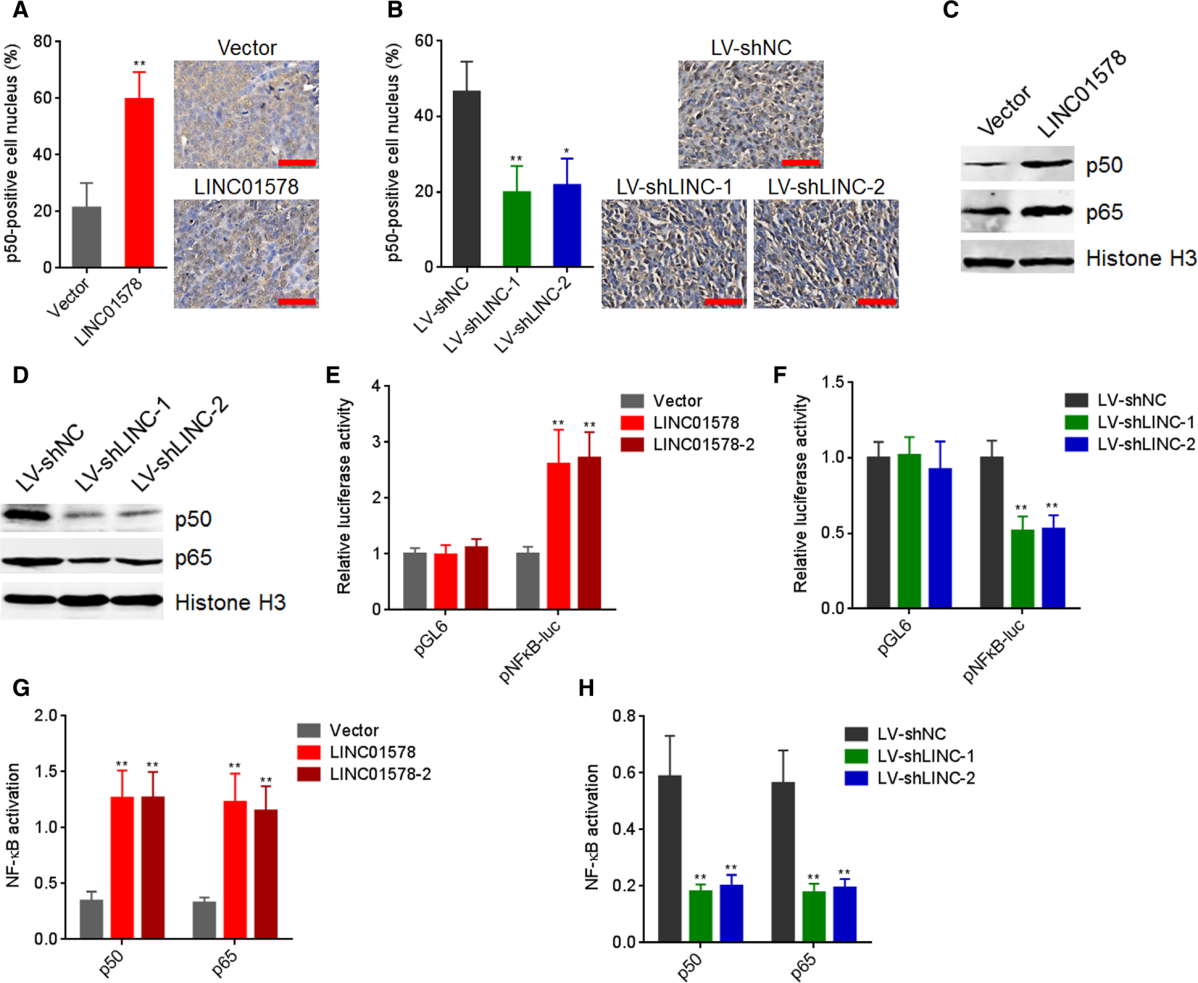
LINC01578 activates NF‐κB signaling. (A) p50 IHC staining of liver metastatic tumors derived from LINC01578‐overexpressed and control DLD‐1 cells. Scale bars, 50 µm. (B) p50 IHC staining of liver metastatic tumors derived from LINC01578‐depleted and control LoVo cells. Scale bars, 50 µm. (C) Nuclear p50 and p65 levels in LINC01578‐overexpressed and control DLD‐1 cells were detected by western blot. (D) Nuclear p50 and p65 levels in LINC01578‐depleted and control LoVo cells were detected by western blot. (E) Firefly luciferase reporters containing NF‐κB binding sites (pNFκB‐luc) were transfected into LINC01578‐overexpressed and control DLD‐1 cells. Then, luciferase reporter assays were performed to determine NF‐κB transcriptional activity. (F) Firefly luciferase reporters containing NF‐κB binding sites (pNFκB‐luc) were transfected into LINC01578‐depleted and control LoVo cells. Then, luciferase reporter assays were performed to determine NF‐κB transcriptional activity. (G) p50 and p65 activation in nuclear extracts from LINC01578‐overexpressed and control DLD‐1 cells was determined by NF‐κB p50 Transcription Factor Assay Kit and NF‐κB p65 Transcription Factor Assay Kit, respectively. (H) p50 and p65 activation in nuclear extracts from LINC01578‐depleted and control LoVo cells was determined by NF‐κB p50 Transcription Factor Assay Kit and NF‐κB p65 Transcription Factor Assay Kit, respectively. Data are shown as mean ± SD based on *n* = 6 mice in each group (A,B) or three independent experiments (C–H). *P < 0.05, **P < 0.01 by the Mann–Whitney test (A), Kruskal–Wallis test followed by Dunn's multiple comparisons test (B), or one‐way ANOVA followed by Dunnett's multiple comparisons test (E–H).

Considering NF‐κB could activate LINC01578, we next studied whether LINC01578 was implicated in NF‐κB signaling in colon cancer. p50 nuclear expression was measured to determine NF‐κB activation in liver metastatic tumors formed by LINC01578‐overexpressed DLD‐1 and LINC01578‐depleted LoVo cells. As presented in Fig. 6A,B, the liver metastatic tumors formed by LINC01578‐overexpressed DLD‐1 cells had higher p50 nuclear expression with respect to the liver metastatic tumors formed by control DLD‐1 cells (Fig. 6A). Conversely, the liver metastatic tumors formed by LINC01578‐depleted LoVo cells had lower p50 nuclear expression with respect to the liver metastatic tumors formed by control LoVo cells (Fig. 6B). Next, NF‐κB nuclear expression in LINC01578‐overexpressed DLD‐1 and LINC01578‐depleted LoVo cells was measured by western blot. LINC01578‐overexpressed DLD‐1 cells had significantly higher p50 and p65 nuclear expression with respect to control DLD‐1 cells (Fig. 6C). Conversely, LINC01578‐depleted LoVo cells had significantly lower p50 and p65 nuclear expression with respect to control LoVo cells (Fig. 6D). To further investigate whether LINC01578 modulated NF‐κB, NF‐κB transcriptional activity was measured using luciferase reporter assays. As shown in Fig. 6E,F, NF‐κB transcriptional activity increased after LINC01578 overexpression and reduced after LINC01578 depletion. NF‐κB activation in nuclear extracts was measured by NF‐κB Transcription Factor Assay. As shown in Fig. 6G,H, p50 and p65 activation in nuclear extracts increased after LINC01578 overexpression and reduced after LINC01578 depletion. Therefore, these findings demonstrated that LINC01578 activated NF‐κB in colon cancer.

**Fig. 7:**
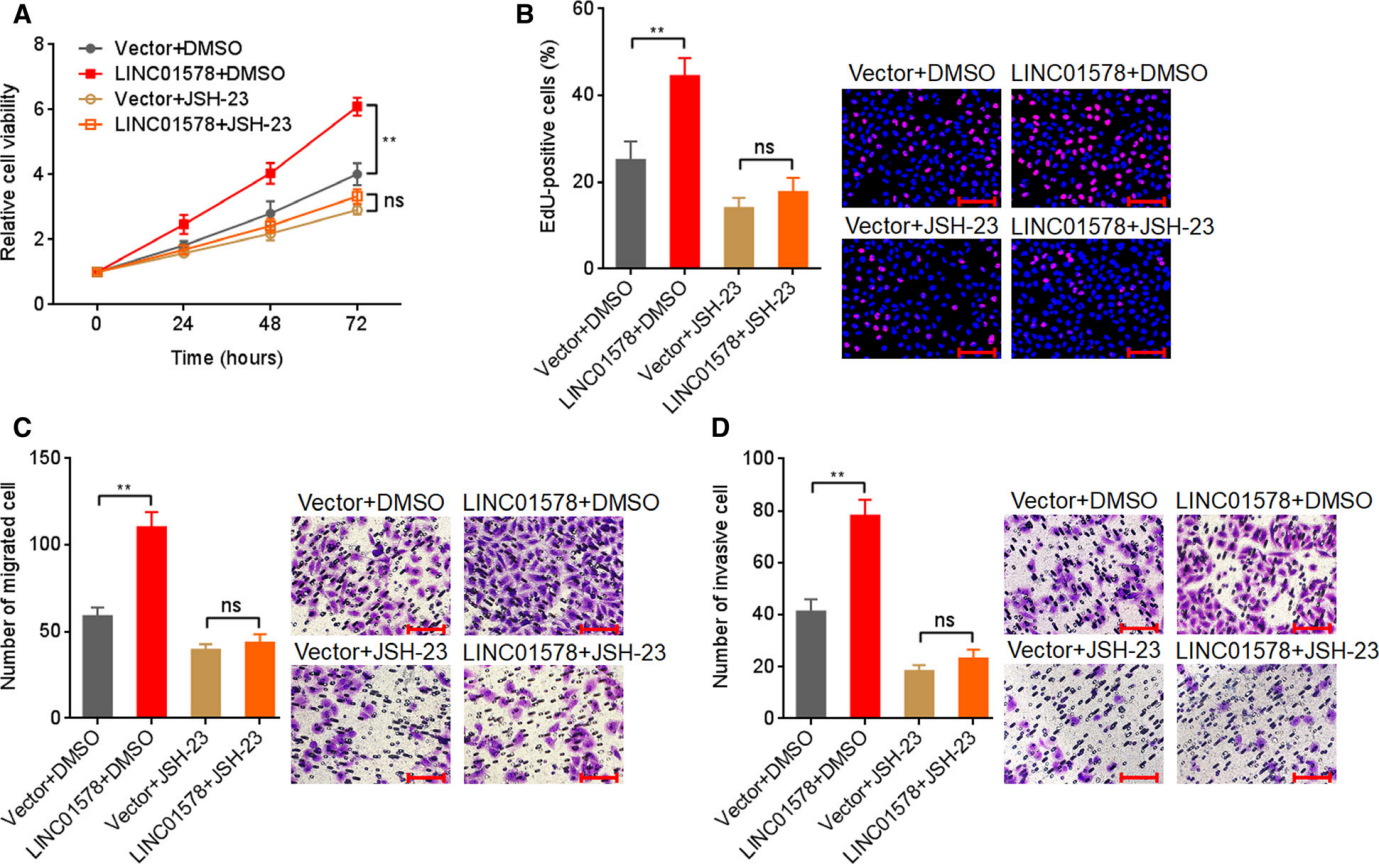
Blocking of NF‐κB signaling abolished the oncogenic roles of LINC01578 in colon cancer. (A) Cell viability of LINC01578 overexpressed and control DLD‐1 cells treated with DMSO or 5 µm JSH‐23 was determined by Glo cell viability assay. (B) Cell proliferation of LINC01578‐overexpressed and control DLD‐1 cells treated with DMSO or 5 µm JSH‐23 was determined by EdU assays. Scale bars, 100 µm. (C) Cell migration of LINC01578‐overexpressed and control DLD‐1 cells treated with DMSO or 5 µm JSH‐23 was determined by transwell migration assays. Scale bars, 100 µm. (D) Cell invasion of LINC01578‐overexpressed and control DLD‐1 cells treated with DMSO or 5 µm JSH‐23 was determined by transwell invasion assays. Scale bars, 100 µm. Data are shown as mean ± SD based on three independent experiments. **P < 0.01, ns, not significant, by Student's *t*‐test.

To elucidate whether the activation of NF‐κB mediated the oncogenic roles of LINC01578 in colon cancer, DLD‐1 cells with LINC01578 overexpression or control were treated with NF‐κB inhibitor JSH‐23. Glo cell viability assays presented that JSH‐23 abolished the roles of LINC01578 overexpression in increasing cell viability (Fig. 7A). EdU assays showed that JSH‐23 abolished the roles of LINC01578 overexpression in accelerating cell proliferation (Fig. 7B). Transwell migration assays presented that JSH‐23 abolished the roles of LINC01578 overexpression in promoting cell migration (Fig. 7C). Transwell invasion assays showed that JSH‐23 abolished the roles of LINC01578 overexpression in promoting cell invasion (Fig. 7D). Collectively, the data demonstrated that LINC01578 enhanced colon cancer cell viability and mobility via activating NF‐κB.

### 3.6 LINC01578 epigenetically repressed IκBβ via recruiting EZH2

**Fig. 8:**
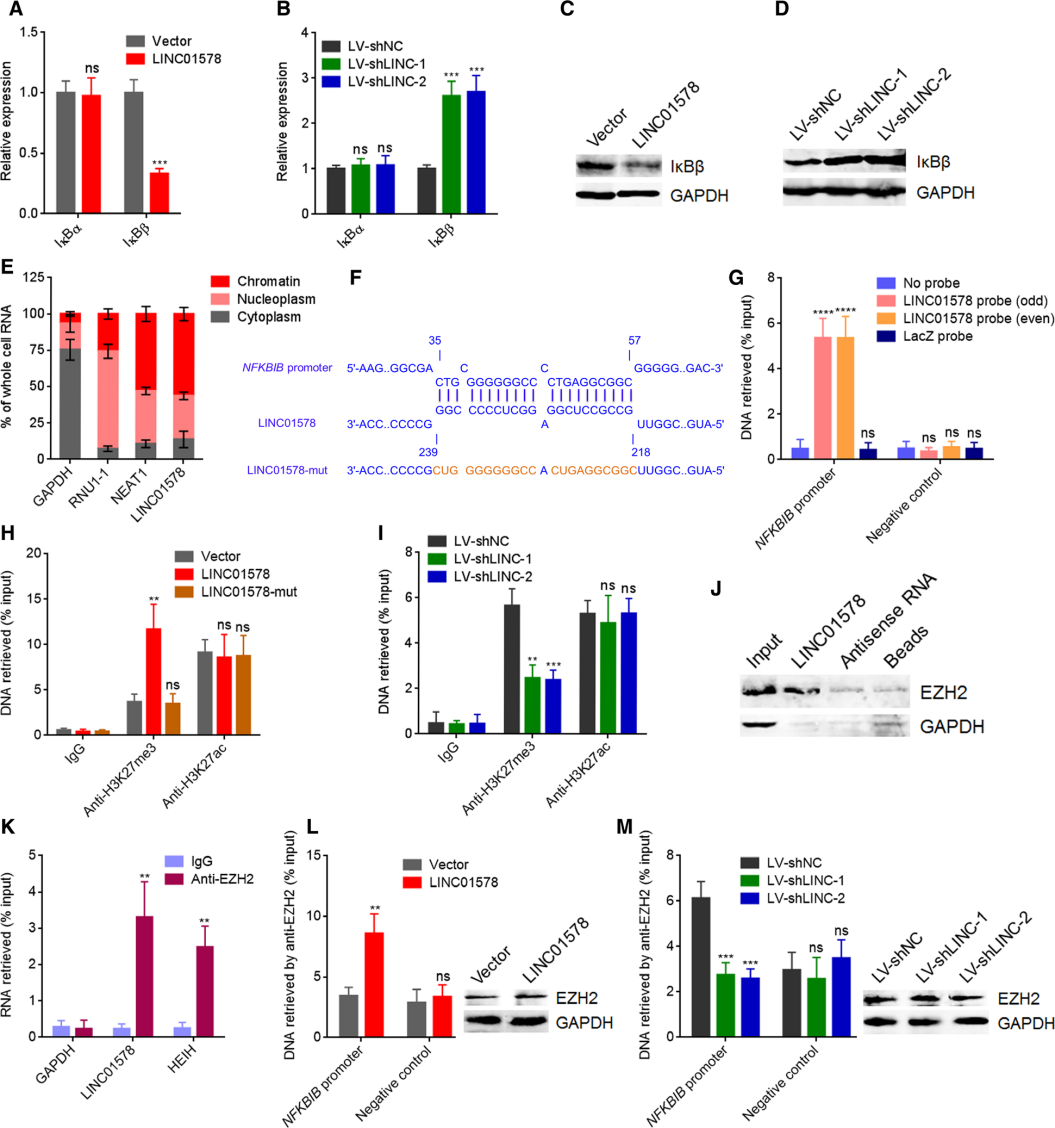
LINC01578 represses IκBβ via binding and recruiting EZH2. (A) IκBα and IκBβ mRNA levels in DLD‐1 cells transfected with LINC01578 overexpression vector were measured by qRT‐PCR. (B) IκBα and IκBβ mRNA levels in LoVo cells infected with shRNAs targeted to LINC01578 were measured by qRT‐PCR. (C) IκBβ protein level in DLD‐1 cells transfected with LINC01578 overexpression vector was measured by western blot. (D) IκBβ protein level in LoVo cells infected with shRNAs targeted to LINC01578 was measured by western blot. (E) Subcellular localization of LINC01578 was detected by qRT‐PCR in biochemically fractionated DLD‐1 cells. GAPDH, RNU1‐1, and NEAT1 were used as cytoplasmic, nucleoplasmic, and chromatinic controls, respectively. (F) The predicted interaction between LINC01578 and *NFKBIB* promoter. (G) ChIRP assays using LINC01578 capture probes were carried out in DLD‐1 cells. The enrichment of *NFKBIB* promoter and a distant NC region was determined by qPCR. (H) ChIP assays using H3K27me3 and H3K27ac antibodies were carried out in DLD‐1 cells overexpressing wide‐type or binding region‐mutated LINC01578. The enrichment of *NFKBIB* promoter was determined by qPCR. (I) ChIP assays using H3K27me3 and H3K27ac antibodies were carried out in LINC01578‐depleted and control LoVo cells. The enrichment of *NFKBIB* promoter was determined by qPCR. (J) RNA pull‐down assays were performed using *in vitro*‐transcribed biotinylated LINC01578. The enriched proteins were detected by western blot. GAPDH was used as NC. (K) RIP assays using EZH2 antibody were carried out in DLD‐1 cells. The enrichment of LINC01578 was determined by qRT‐PCR. GAPDH and HEIH were used as negative and positive controls, respectively. (L) ChIP assays using EZH2 antibody were carried out in LINC01578‐overexpressed and control DLD‐1 cells. The enrichment of *NFKBIB* promoter and a distant NC region was determined by qPCR. EZH2 protein level in LINC01578‐overexpressed and control DLD‐1 cells was determined by western blot. (M) ChIP assays using EZH2 antibody were carried out in LINC01578‐depleted and control LoVo cells. The enrichment of *NFKBIB* promoter and a distant NC region was determined by qPCR. EZH2 protein level in LINC01578‐depleted and control LoVo cells was determined by western blot. Data are shown as mean ± SD based on three independent experiments. **P < 0.01, ***P < 0.001, ****P < 0.0001, ns, not significant, by Student's *t*‐test (A,K,L), or one‐way ANOVA followed by Dunnett's multiple comparisons test (B,G,H,I,M).

To investigate the mechanisms mediating the activation of NF‐κB by LINC01578, LINC01578‐overexpressed and control DLD‐1 cells were treated with different NF‐κB inhibitors, and then, NF‐κB activation in nuclear extracts was measured by NF‐kB Transcription Factor Assay. NF‐κB nuclear translocation inhibitor JSH‐23 abolished the increased NF‐κB activation caused by LINC01578 (Fig. S5A). IKK inhibitor Bay 11‐7085 did not influence the increased NF‐κB activation caused by LINC01578 (Fig. S5B). Therefore, the targets of LINC01578 were locating at the downstream of IKK and upstream of p50/p65. Next, we investigated the effects of LINC01578 on IκBα and IκBβ. As shown in Fig. 8A–D, IκBβ expression decreased after LINC01578 overexpression and increased after LINC01578 depletion. Furthermore, the liver metastatic tumors formed by LINC01578‐overexpressed DLD‐1 cells had lower IκBβ expression with respect to the liver metastatic tumors formed by control DLD‐1 cells (Fig. S5C). The liver metastatic tumors formed by LINC01578‐depleted LoVo cells had increased IκBβ expression with respect to the liver metastatic tumors formed by control LoVo cells (Fig. S5D). The data further supported the repressive roles of LINC01578 on IκBβ.

To study the mechanisms mediating the repressive roles of LINC01578 on IκBβ, we determined the subcellular localization of LINC01578. As shown in Fig. 8E, LINC01578 mainly localizes at chromatin. Next, we predicted a putative LINC01578 complementary binding locus in *NFKBIB* (IκBβ encoding gene) promoter (Fig. 8F). ChIRP assay presented the specific binding of LINC01578 to *NFKBIB* promoter, but not a distant NC region (Fig. 8G). To elucidate whether the binding between LINC01578 and *NFKBIB* promoter modulated chromatin structure, chromatin marks at *NFKBIB* promoter region were investigated using ChIP assay. As shown in Fig. 8H, repressive chromatin mark H3K27me3 significantly increased after LINC01578 overexpression, which was abolished by the mutation of the binding region. Furthermore, H3K27me3 significantly reduced after LINC01578 depletion (Fig. 8I). H3K27me3 was mainly catalyzed by histone methyltransferase EZH2. Intriguingly, the online *in silico* tool RPISeq (http://pridb.gdcb.iastate.edu/RPISeq/) predicted a potential binding between LINC01578 and EZH2 with a score of 0.96. RNA pull‐down assay presented specific enrichment of EZH2 in the LINC01578 group (Figs 8J and S5E). RIP assays showed specific enrichment of LINC01578 in the EZH2 antibody group (Fig. 8K). EZH2‐interacted lncRNA HEIH was used as positive control [40]. Thus, the RNA pull‐down and RIP assays suggested the specific binding between LINC01578 and EZH2. ChIP assays further demonstrated that the binding of EZH2 to *NFKBIB* promoter increased after LINC01578 overexpression and reduced after LINC01578 depletion, while EZH2 levels were not changed by LINC01578 (Fig. 8L,M). Collectively, these results demonstrated that LINC01578 binds and recruits EZH2 to *NFKBIB* promoter, and therefore epigenetically represses LINC01578 expression.

### 3.7 LINC01578 formed a positive feedback loop with NF‐κB/YY1

**Fig. 9:**
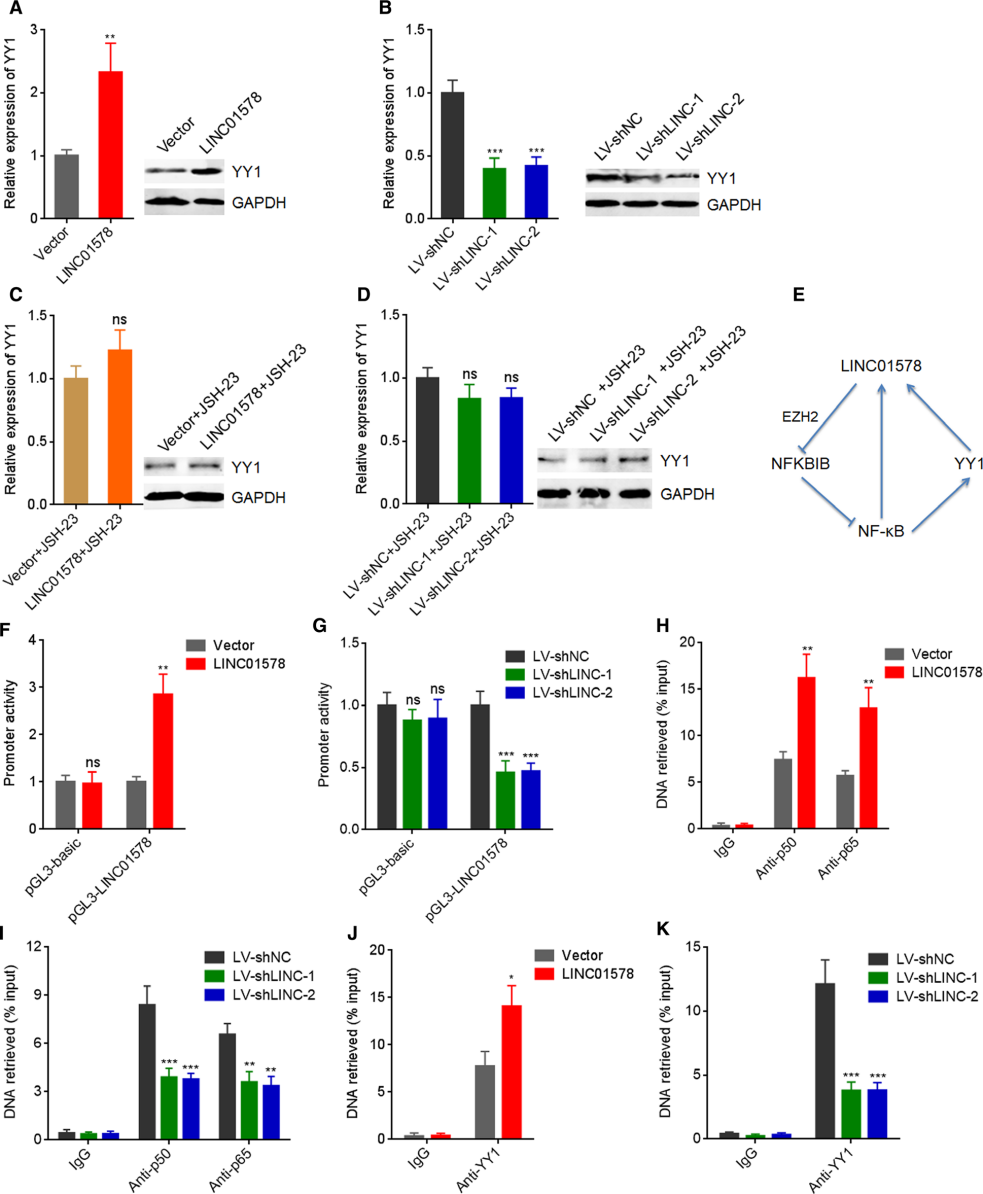
LINC01578 forms a positive feedback loop with NF‐κB/YY1. (A) YY1 mRNA and protein levels in DLD‐1 cells transfected with LINC01578 overexpression vector were determined by qRT‐PCR and western blot. (B) YY1 mRNA and protein levels in LoVo cells infected with shRNAs targeted to LINC01578 were determined by qRT‐PCR and western blot. (C) YY1 mRNA and protein levels in DLD‐1 cells transfected with LINC01578 overexpression vector and treated with 5 µm JSH‐23 were determined by qRT‐PCR and western blot. (D) YY1 mRNA and protein levels in LoVo cells infected with shRNAs targeted to LINC01578 and treated with 5 µm JSH‐23 were determined by qRT‐PCR and western blot. (E) A schematic model of positive feedback loop between LINC01578 and NF‐κB/YY1. (F) Luciferase reporter assays for LINC01578‐overexpressed and control DLD‐1 cells transfected with luciferase reporter plasmids containing *LINC01578* promoter. (G) Luciferase reporter assays for LINC01578‐depleted and control LoVo cells transfected with luciferase reporter plasmids containing *LINC01578* promoter. (H) ChIP assays using p50 and p65 antibodies were carried out in LINC01578‐overexpressed and control DLD‐1 cells. The enrichment of *LINC01578* promoter was determined by qPCR. (I) ChIP assays using p50 and p65 antibodies were carried out in LINC01578‐depleted and control LoVo cells. The enrichment of *LINC01578* promoter was determined by qPCR. (J) ChIP assays using YY1 antibody were carried out in LINC01578‐overexpressed and control DLD‐1 cells. The enrichment of *LINC01578* promoter was determined by qPCR. (K) ChIP assays using YY1 antibody were carried out in LINC01578‐depleted and control LoVo cells. The enrichment of *LINC01578* promoter was determined by qPCR. Data are shown as mean ± SD based on three independent experiments. *P < 0.05, **P < 0.01, ***P < 0.001, ns, not significant, by Student's *t*‐test (A,C,F,H,J), or one‐way ANOVA followed by Dunnett's multiple comparisons test (B,D,G,I,K).

Yin Yang 1 was frequently reported as downstream target of NF‐κB in several different cell models [39, 41, 42]. In the above‐established liver metastasis models, we found that the liver metastatic tumors formed by LINC01578‐overexpressed DLD‐1 cells had higher YY1 expression with respect to the liver metastatic tumors formed by control DLD‐1 cells (Fig. S5F). The liver metastatic tumors formed by LINC01578‐depleted LoVo cells had lower YY1 expression with respect to the liver metastatic tumors formed by control LoVo cells (Fig. S5G). To further confirm whether LINC01578 regulates YY1 through NF‐κB in colon cancer, YY1 expression in LINC01578‐overexpressed DLD‐1 and LINC01578‐depleted LoVo cells was detected by qRT‐PCR and western blot. As presented in Fig. 9A,B, YY1 expression increased after LINC01578 overexpression and reduced after LINC01578 depletion. Furthermore, both the increase in YY1 caused by LINC01578 overexpression and reduction in YY1 caused by LINC01578 depletion were abolished by JSH‐23 (Fig. 9C,D). These results suggested that LINC01578 upregulated YY1 through activation of NF‐κB. Given that NF‐κB and YY1 directly upregulated LINC01578, LINC01578 and NF‐κB/YY1 may form positive feedback loop in colon cancer (Fig. 9E). To verify whether this feedback loop existed, pGL3 luciferase reporter plasmid assay was conducted to detect the effects of LINC01578 on *LINC01578* promoter activity. As presented in Fig. 9F,G, *LINC01578* promoter activity increased after LINC01578 overexpression and reduced after LINC01578 depletion. Furthermore, the binding of NF‐κB on *LINC01578* promoter increased after LINC01578 overexpression and reduced after LINC01578 depletion (Fig. 9H,I). Consistently, the binding of YY1 on *LINC01578* promoter increased after LINC01578 overexpression and reduced after LINC01578 depletion (Fig. 9J,K). Therefore, these findings demonstrated a positive feedback loop between LINC01578 and NF‐κB/YY1.

**Fig. 10:**
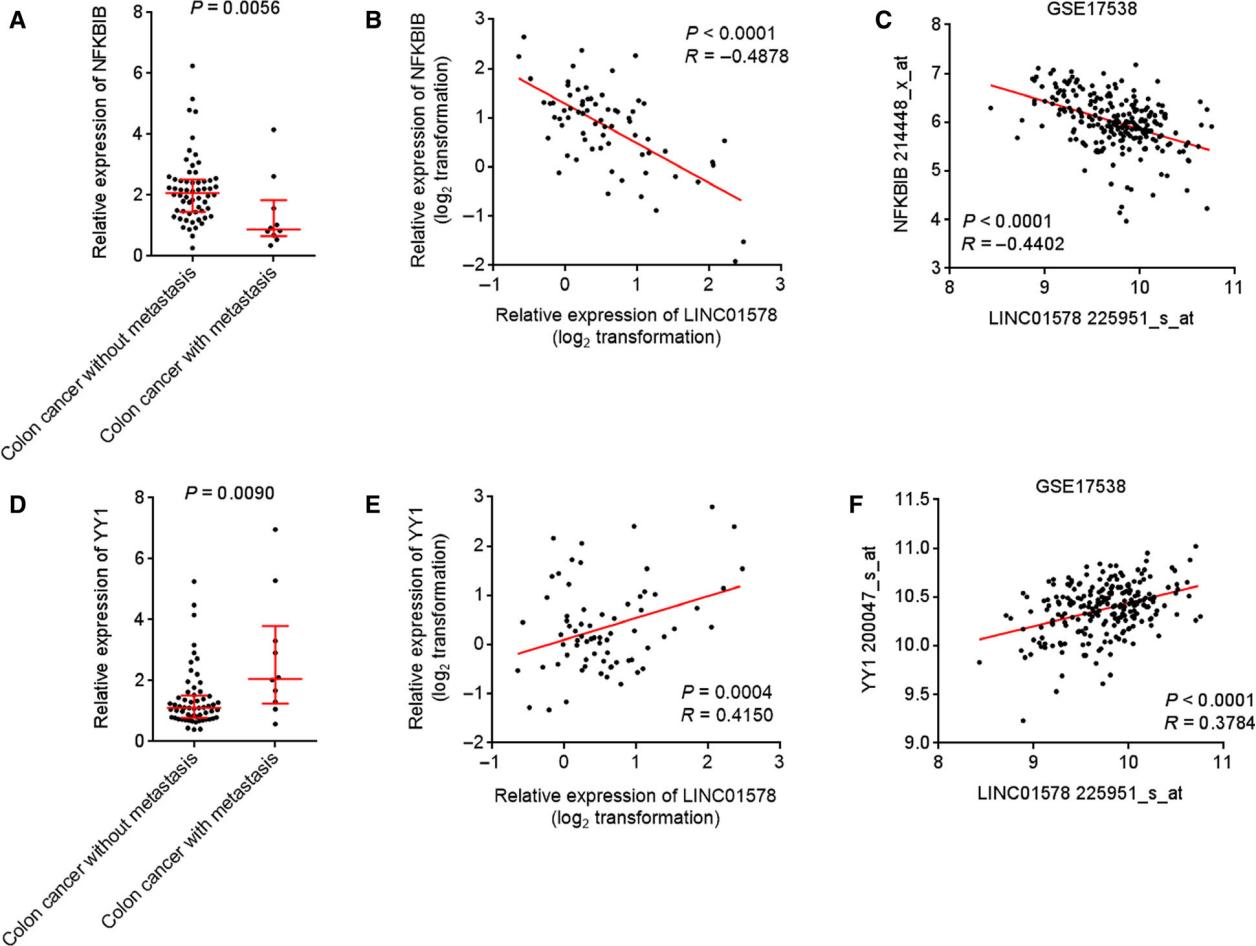
The expression pattern and correlations of LINC01578/NFKBIB/YY1 in colon cancer liver metastasis. (A) NFKBIB expression in 60 colon cancer tissues without metastasis and 10 colon cancer tissues with metastasis was measured by qRT‐PCR. *P* = 0.0056 by the Mann–Whitney test. (B) The correlation between NFKBIB and LINC01578 expression in these 70 colon cancer tissues. *P* < 0.0001, *R* = −0.4878 by Pearson's correlation analysis. (C) The correlation between NFKBIB and LINC01578 expression in GSE17538 dataset. *P* < 0.0001, *R* = −0.4402 by Pearson's correlation analysis. (D) YY1 expression in 60 colon cancer tissues without metastasis and 10 colon cancer tissues with metastasis was measured by qRT‐PCR. *P* = 0.0090 by the Mann–Whitney test. (E) The correlation between YY1 and LINC01578 expression in these 70 colon cancer tissues. *P* = 0.0004, *R* = 0.4150 by the Pearson correlation analysis. (F) The correlation between YY1 and LINC01578 expression in GSE17538 dataset. *P* < 0.0001, *R* = 0.3784 by the Pearson correlation analysis.

To investigate whether this positive feedback loop also exists in colon cancer metastasis *in vivo*, we measured the expression and correlation between LINC01578, NFKBIB, and YY1 in clinical tissues. In contrast to LINC01578, NFKBIB was remarkably downregulated in colon cancer tissues with metastasis with respect to colon cancer tissues without metastasis (Fig. 10A). The expression of NFKBIB was remarkably inversely correlated with that of LINC01578 in colon cancer tissues (Fig. 10B). The inverse correlation between NFKBIB and LINC01578 was also found in GSE17538 dataset (Fig. 10C). Consistent with LINC01578, YY1 was also increased in colon cancer tissues with metastasis with respect to colon cancer tissues without metastasis (Fig. 10D). The expression of YY1 was positively correlated with that of LINC01578 in colon cancer tissues (Fig. 10E). The positive correlation between YY1 and LINC01578 was also found in GSE17538 dataset (Fig. 10F). These findings supported the positive feedback loop between LINC01578 and NF‐κB/YY1 in human colon cancer metastasis.

## 4 Discussion

Metastasis accounts for the majority of cancer‐related death [43, 44]. Identifying the aberrantly expressed molecules and critical molecular events during metastasis would provide prognostic biomarker and therapeutic target for metastasis [45]. In this study, using publicly available dataset including TCGA and GEO data, and our own cohort collected in our hospital, we identified lncRNA LINC01578 as a metastasis and prognosis correlated with lncRNA in colon cancer. Our findings identified that increased expression of LINC01578 was correlated with worse overall survival, DSS, and DFS. Further investigation on multiple central cohorts is needed to establish the optimal cutoff value of LINC01578 for potential clinical application to predict the outcomes of colon cancer patients. Furthermore, our findings revealed that LINC01578 was increased in colon cancer tissues with respect to normal tissues. Increased expression of LINC01578 was positively associated with lymph node metastasis, distant metastasis, and advanced clinical stage. The expression of LINC01578 was increased in colon cancer tissues with metastasis with respect to colon cancer tissues without metastasis. However, LINC01578 was not differentially expressed between primary colon cancer tissues and matched liver metastatic tissues. Therefore, our findings suggest that different LINC01578 expressions in primary colon cancer tissues may determine different metastatic potential of corresponding colon cancer.

Gain‐of‐function and loss‐of‐function assays demonstrated that ectopic expression of LINC01578 enhanced colon cancer cell viability and mobility *in vitro* and colon cancer liver metastasis *in vivo*. Depletion of LINC01578 inhibited colon cancer cell viability and mobility *in vitro* and colon cancer liver metastasis *in vivo*. Further analysis of the liver metastatic lesions revealed that LINC01578 promoted colon cancer cell proliferation and repressed colon cancer cell apoptosis *in vivo*. Therefore, our findings suggested that LINC01578 drove colon cancer liver metastasis via enhancing colon cancer cell viability and mobility. Our findings also suggested LINC01578 as a potential therapeutic target for colon cancer liver metastasis.

The mechanisms exerted by lncRNAs are complicated [46, 47, 48]. Different subcellular locations of lncRNAs influence their mechanisms of action. Cytoplasmic lncRNAs interact and sponge microRNAs, and relieve the repressive roles of microRNAs on their targets [49]. Cytoplasmic lncRNAs also bind mRNAs and change the stability and/or translation of interacted mRNAs [50]. Nuclear lncRNAs may regulate the transcription of target genes via binding epigenetic modification enzymes or transcription factors [51]. Chromatin‐bound lncRNAs may directly change the epigenetic modification and chromatin structure, and further modulate target gene expression [52]. Another important common mechanism of lncRNAs is binding proteins [53, 54]. The interaction between lncRNAs and proteins may regulate the post‐translational modification, location, expression, and/or role of the bound proteins.

Here, we identified LINC01578 as a chromatin‐bound lncRNA, and further found that LINC01578 specifically binds *NFKBIB* promoter. *NFKBIB* encodes IκBβ, an important inhibitor of NF‐κB. Combining bioinformatic analyses and experimental investigation, we further revealed that LINC01578 bound and recruited EZH2 to *NFKBIB* promoter, upregulated H3K27me3 level in *NFKBIB* promoter, and therefore repressed *NFKBIB* expression. Via repressing *NFKBIB* expression, LINC01578 activated NF‐κB signaling. NF‐κB signaling was frequently reported to play critical roles in cancer cell viability and tumor growth and metastasis [55, 56]. Here, we further found that blocking NF‐κB signaling abolished the oncogenic roles of LINC01578 in colon cancer, suggesting that NF‐κB signaling activation was an important mediator of the oncogenic roles of LINC01578 in colon cancer. NF‐κB family contains five different transcription factors, including p65, p50/p105, p52/p100, RelB, and c‐Rel. Here, we focused on the canonical p65 and p50. The effects of LINC01578 on noncanonical NF‐κB signaling need further study. The identification of LINC01578 as a chromatin‐bound lncRNA provided novel evidence for the functional variety of lncRNAs. lncRNAs NEAT1 and MALAT1 were revealed to bind active chromatin sites [52]. But the effects of NEAT1 and MALAT1 on the binding sites are not clear [52]. lncRNA DILC was shown to bind *IL‐6* promoter and inhibited *IL‐6* transcription [57]. lncRNA RBAT1 bound to *E2F3* promoter and activated *E2F3* transcription [58]. These reports demonstrated that the binding to chromatin by lncRNAs may further cause different effects on their bound targets.

Yin Yang 1 is a multifunctional transcription factor, which could positively or negatively regulate target gene expression [59, 60]. YY1 was also reported to be a downstream target of NF‐κB in myoblasts and pancreatic cancer cells [39, 41]. Here, we further found that via activating NF‐κB, LINC01578 upregulated YY1 expression, which was abolished by NF‐κB inhibitor. Intriguingly, LINC01578 was also identified as a direct transcriptional target of NF‐κB and YY1. Our findings revealed that both NF‐κB and YY1 activated *LINC01578* promoter activity and expression. Therefore, LINC01578 formed positive feedback loop with NF‐κB/YY1. Through this positive feedback loop, LINC01578 activated its own promoter activity. The correlation between LINC01578 and NF‐κB/YY1 was also verified in clinical colon cancer tissues and colon cancer liver metastasis. Several feedback loops have been reported in cancers [39, 61]. The feedback loop amplifies the effects of the participants and drives the progressively oncogenic and metastatic processes.

## 5 Conclusion

Here, we found lncRNA LINC01578 was correlated with colon cancer metastasis and poor prognosis. LINC01578 enhances colon cancer cell viability, mobility, and *in vivo* metastasis through forming a positive feedback loop with NF‐κB/YY1. LINC01578 represents a valuable prognostic biomarker and therapeutic target for colon cancer metastasis.

## Conflict of interest

The authors declare no conflict of interest.

## Author contributions

DK and JL designed the study. JL, YZ, JW (Jiefu Wang), and JW (Junfeng Wang) performed the experiments. DK, JL, YZ, and JG analyzed and interpreted the data. DK and JL wrote the manuscript.

## Supporting information


**Fig. S1.** The expression and characteristics of LINC01578 in colon cancer.

**Fig. S2.** NF‐κB and YY1 activated LINC01578 expression in HT‐29 cells.

**Fig. S3.** Overexpression of LINC01578 enhanced DLD‐1 cell viability and mobility.

**Fig. S4.** Depletion of LINC01578 repressed HT‐29 cell viability and mobility.

**Fig. S5.** LINC01578 repressed IκBβ and activated YY1.

**Table S1.** The list of genes correlated with overall survival of colon cancer.

**Table S2.** Univariate and multivariate survival analysis of patients with LINC01578 low and high expression.

FigLegends
