# Supplementary figures and images for: Long noncoding RNA LINC01578 drives colon cancer metastasis through a positive feedback loop with the NF‐κB/YY1 axis
Source: Mol Oncol. 2020 Oct 25;14(12):3211–33. doi: 10.1002/1878-0261.12819 (PMC7718957; doi:10.1002/1878-0261.12819)

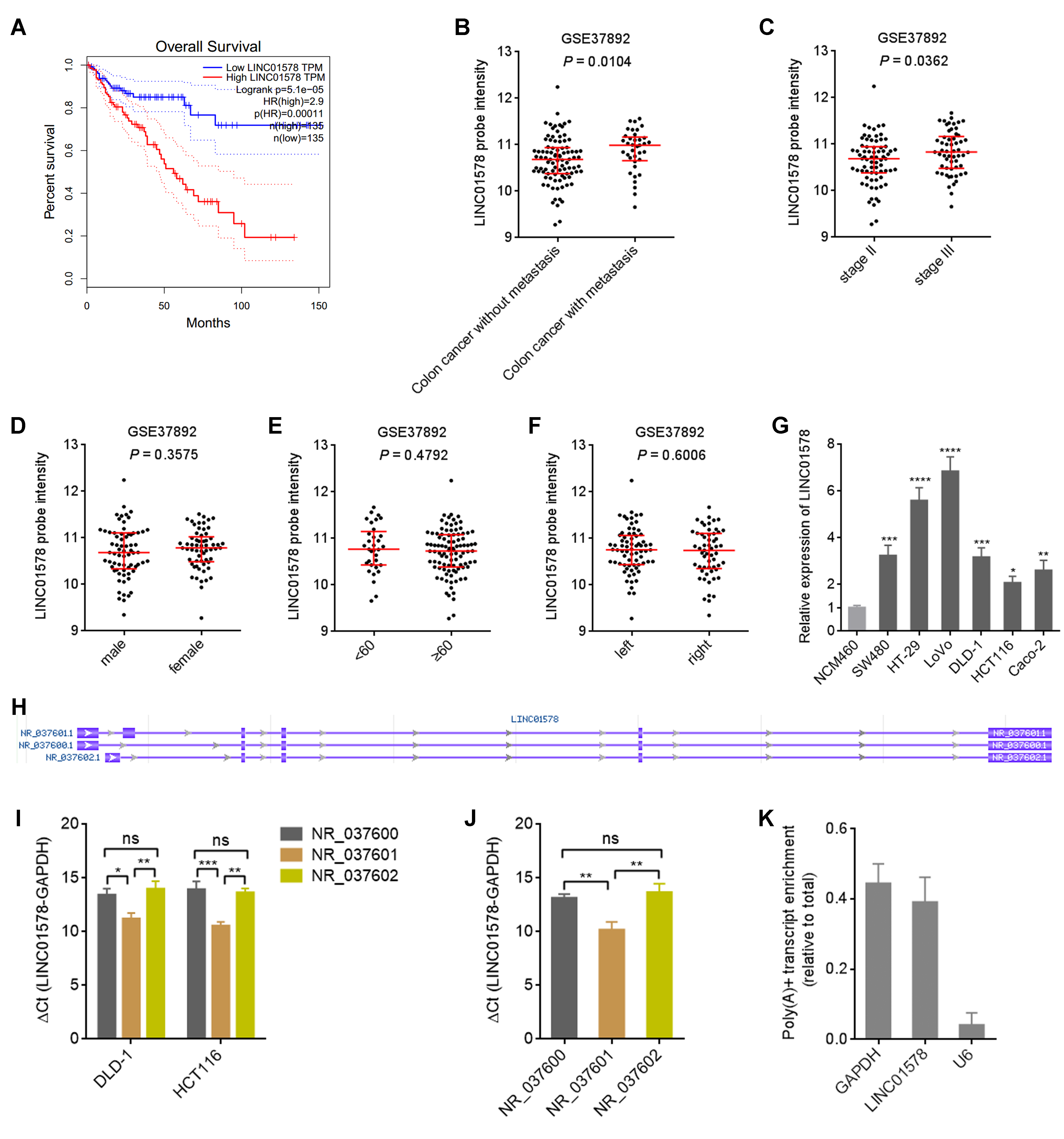

Supplement: Supplementary file 1 — Fig. S1. The expression and characteristics of LINC01578 in colon cancer. [file MOL2-14-3211-s001.tif]

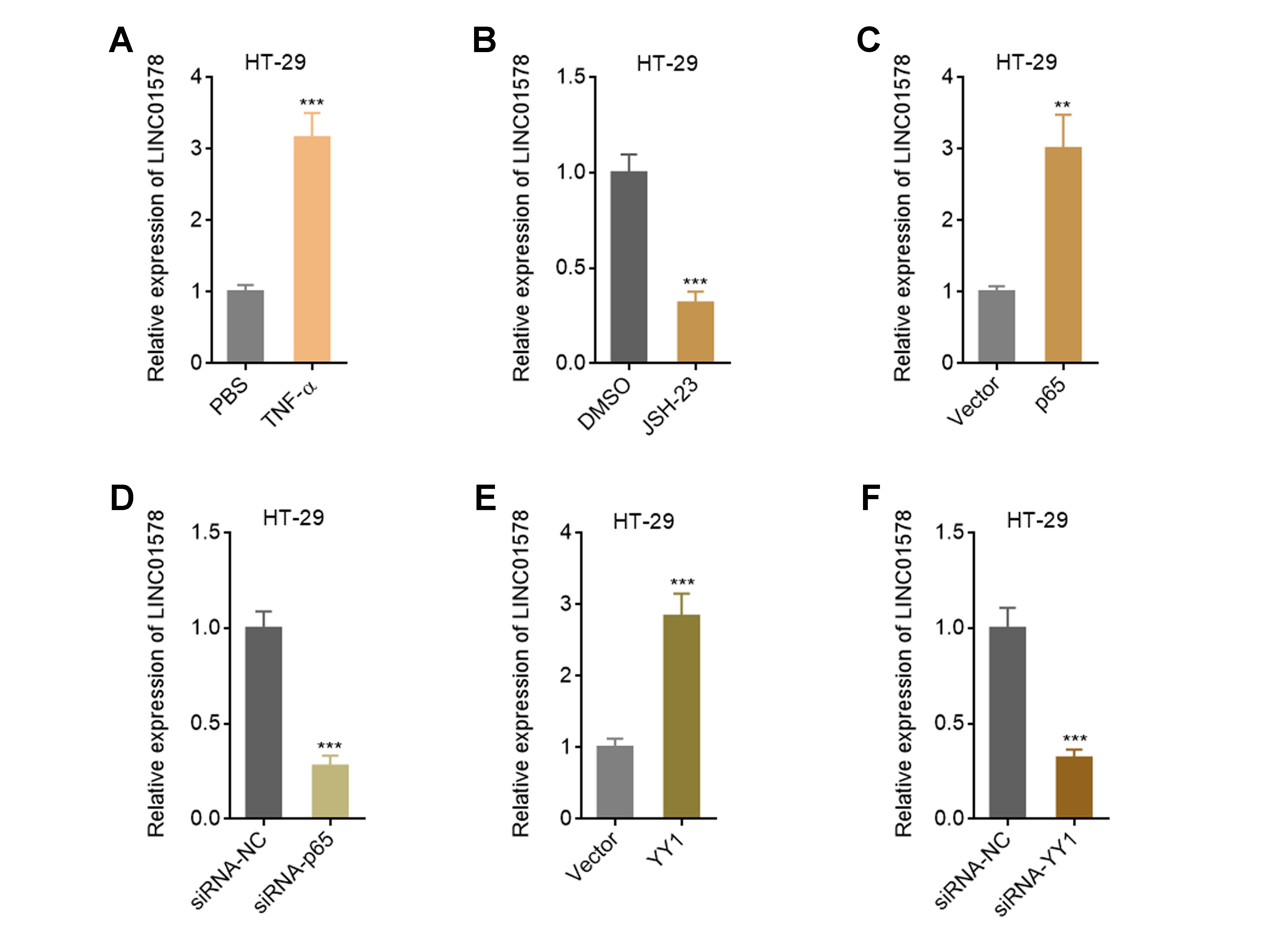

Supplement: Supplementary file 2 — Fig. S2. NF‐κB and YY1 activated LINC01578 expression in HT‐29 cells. [file MOL2-14-3211-s002.tif]

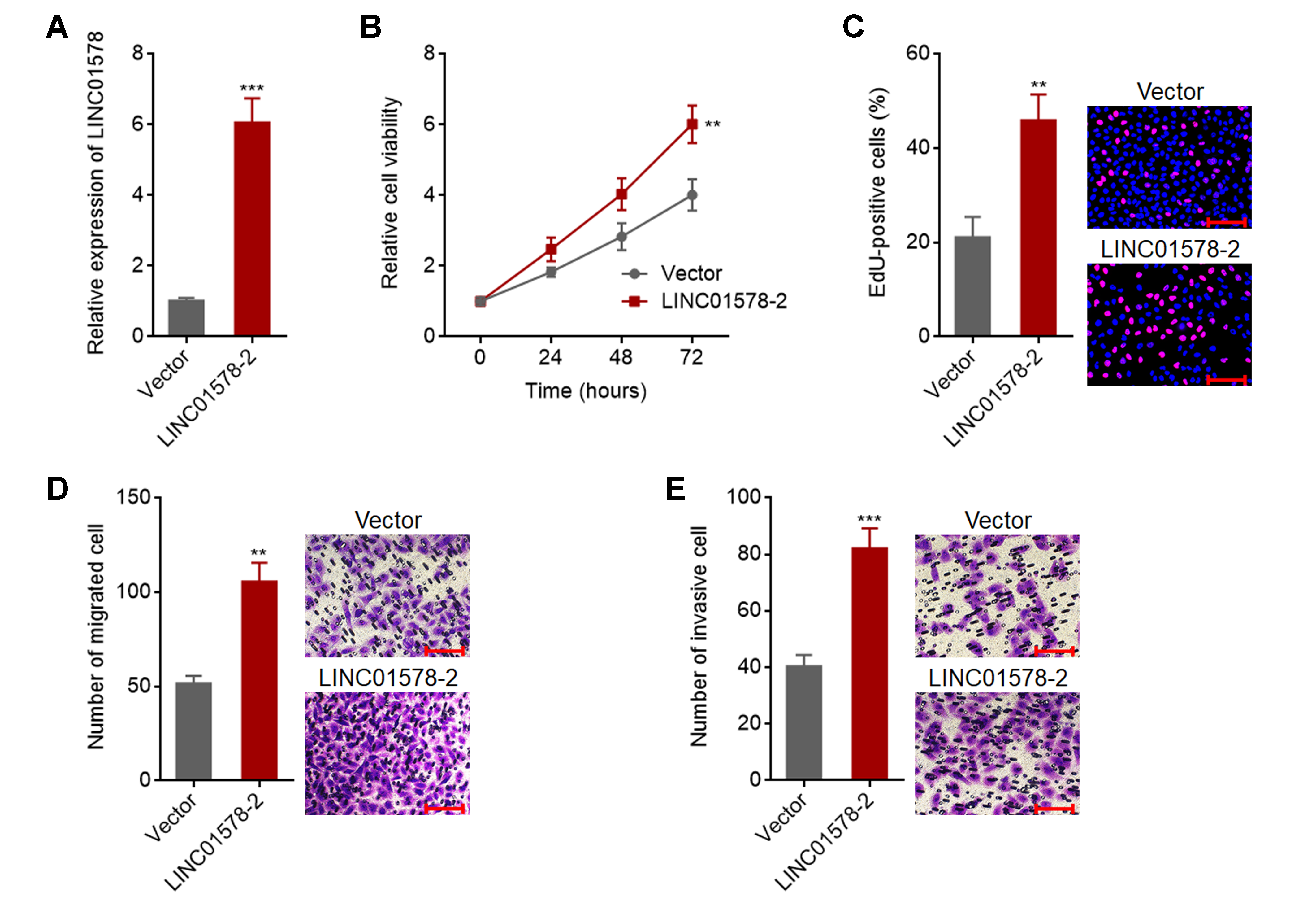

Supplement: Supplementary file 3 — Fig. S3. Overexpression of LINC01578 enhanced DLD‐1 cell viability and mobility. [file MOL2-14-3211-s003.tif]

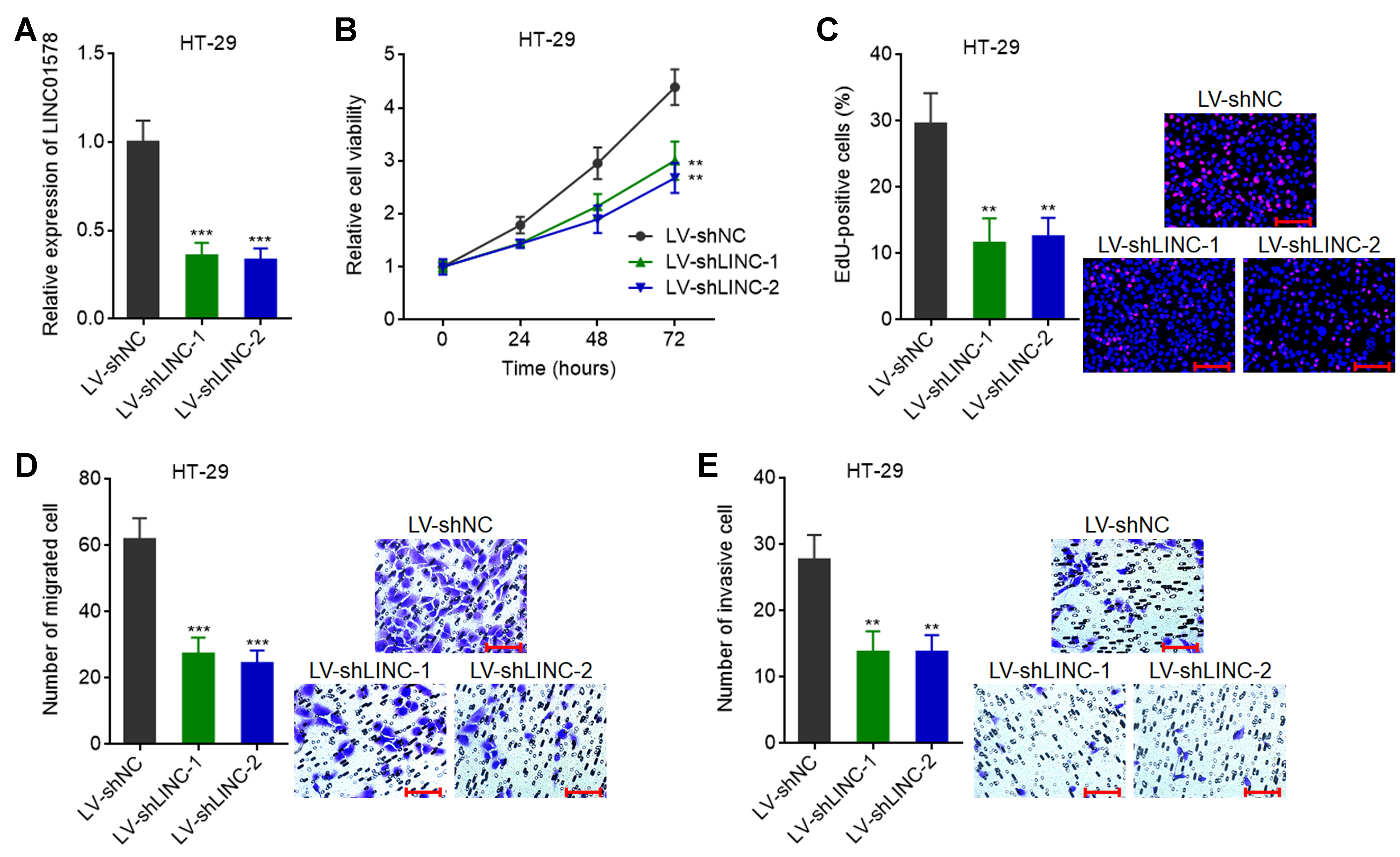

Supplement: Supplementary file 4 — Fig. S4. Depletion of LINC01578 repressed HT‐29 cell viability and mobility. [file MOL2-14-3211-s004.tif]

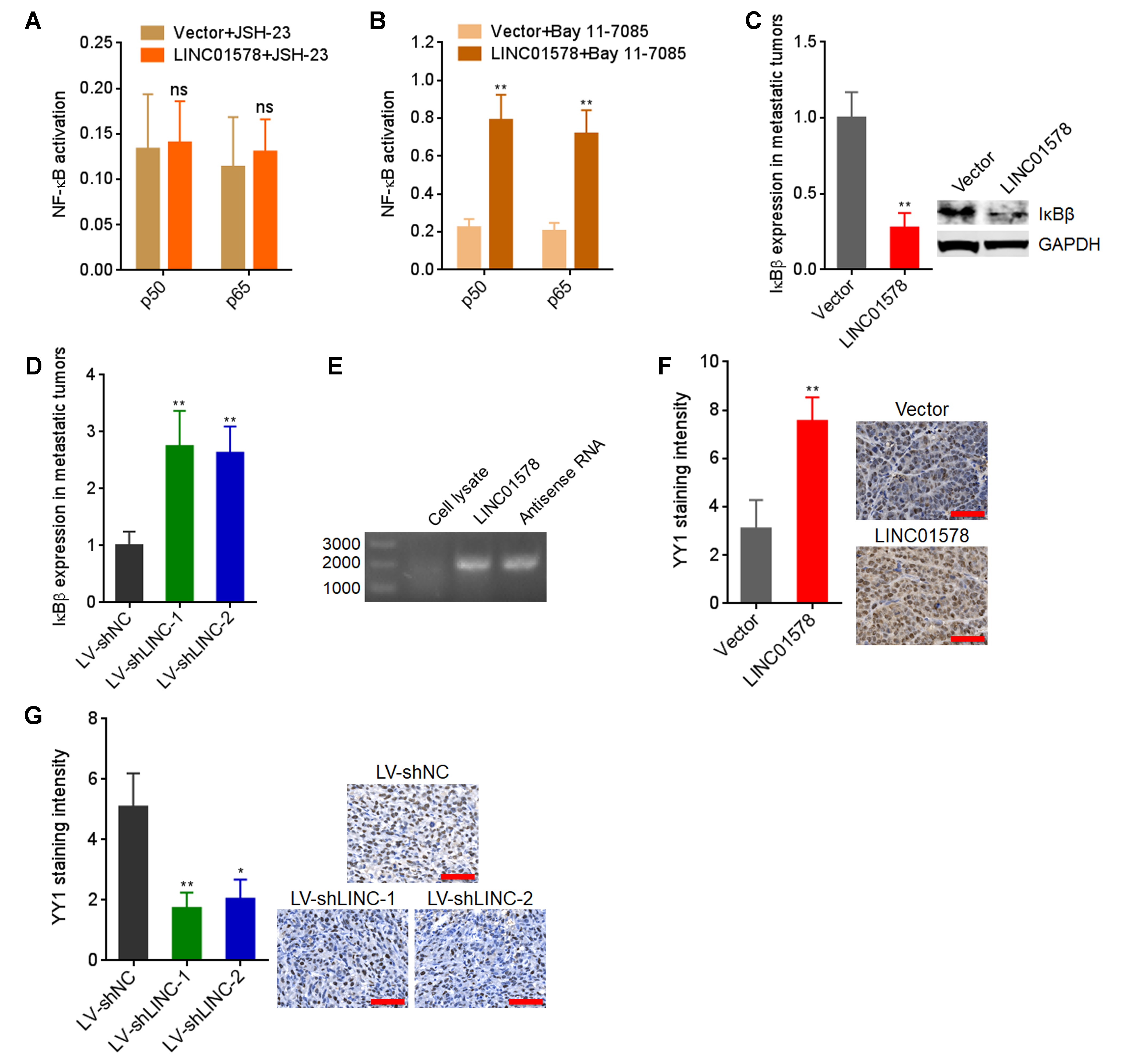

Supplement: Supplementary file 5 — Fig. S5. LINC01578 repressed IκBβ and activated YY1. [file MOL2-14-3211-s005.tif]
