# Supplementary material for: Long noncoding RNA LINC01578 drives colon cancer metastasis through a positive feedback loop with the NF‐κB/YY1 axis
Source: Mol Oncol. 2020 Oct 25;14(12):3211–33. doi: 10.1002/1878-0261.12819 (PMC7718957; doi:10.1002/1878-0261.12819)
Supplement: Supplementary file 7 — Table S2. Univariate and multivariate survival analysis of patients with LINC01578 low and high expression. [file MOL2-14-3211-s007.docx]

**Table S2.** Univariate and multivariate survival analysis of patients with LINC01578 low and high expression.

| Variables | Disease specific survival | |  | Disease free survival | |
| --- | --- | --- | --- | --- | --- |
|  | Hazard ratio (95% CI) | *P* value |  | Hazard ratio (95% CI) | *P* value |
| **Univariate analysis** |  |  |  |  |  |
| LINC01578 (high versus low) | 2.505 (1.055-5.900) | 0.039 |  | 2.109 (1.082-4.307) | 0.031 |
| Gender (female versus male) | 0.816 (0.346-1.919) | 0.642 |  | 0.854 (0.431-1.688) | 0.650 |
| Age (≥60 versus <60) | 0.904 (0.382-2.132) | 0.817 |  | 0.979 (0.493-1.944) | 0.952 |
| Grade (poor versus well/moderate) | 1.134 (0.367-3.547) | 0.820 |  | 1.082 (0.438-2.691) | 0.860 |
| MSI status (MSI verse MSS) | 0.996 (0.402-2.465) | 0.992 |  | 1.078 (0.519-2.250) | 0.837 |
| pT stage (T3-4 versus T1-2) | 2.660 (0.738-5.843) | 0.169 |  | 3.017 (0.995-5.013) | 0.053 |
| pN Stage (N1-2 versus N0) | 1.725 (0.737-4.134) | 0.208 |  | 1.646 (0.839-3.340) | 0.147 |
| pM Stage (M1 versus M0) | 2.688 (1.054-17.34) | 0.043 |  | 1.838 (0.726-6.551) | 0.168 |
| Clinical stage (III-IV versus I-II) | 2.458 (1.032-5.762) | 0.043 |  | 2.032 (1.038-4.117) | 0.040 |
| **Multivariate analysis** |  |  |  |  |  |
| LINC01578 (high versus low) | 2.512 (1.013-6.230) | 0.047 |  | 2.118 (1.052-4.265) | 0.036 |

Univariate survival analysis was calculated by Kaplan-Meier analysis. Multivariate survival analysis was calculated by Cox proportional hazards regression. MSI, microsatellite instability; MSS, microsatellite stability; pT, pathological staging of tumor; pN, pathological staging of lymph node; pM, pathological staging of metastasis.
