# Supplementary material for: Long noncoding RNA LINC01578 drives colon cancer metastasis through a positive feedback loop with the NF‐κB/YY1 axis
Source: Mol Oncol. 2020 Oct 25;14(12):3211–33. doi: 10.1002/1878-0261.12819 (PMC7718957; doi:10.1002/1878-0261.12819)
Supplement: Supplementary file 8 — FigLegends [file MOL2-14-3211-s008.docx]

**Supplementary figure legends**

**Fig. S1.** The expression and characteristics of LINC01578 in colon cancer. (A) The correlation between LINC01578 expression and overall survival in TCGA COAD data was analyzed by GEPIA. (B) LINC01578 expression in 93 colon cancer tissues without metastasis and 37 colon cancer tissues with metastasis from GSE37892 dataset. *P* = 0.0104 by Mann-Whitney test. (C) LINC01578 expression in 73 stage II colon cancer tissues and 57 stage III colon cancer tissues from GSE37892 dataset. *P* = 0.0362 by Mann-Whitney test. (D) LINC01578 expression in 69 male colon cancer tissues and 61 female colon cancer tissues from GSE37892 dataset. *P* = 0.3575 by Mann-Whitney test. (E) LINC01578 expression in 33 <60 years old colon cancer tissues and 97 ≥60 years old colon cancer tissues from GSE37892 dataset. *P* = 0.4792 by Mann-Whitney test. (F) LINC01578 expression in 72 left colon cancer tissues and 57 right colon cancer tissues from GSE37892 dataset. *P* = 0.6006 by Mann-Whitney test. (G) LINC01578 expression in colon cancer cell lines (SW480, HT-29, LoVo, DLD-1, HCT116, and Caco-2) and normal colon cell line (NCM460) was measured by qRT-PCR. Data are shown as mean ± SD based on three independent experiments. **P* < 0.05, ***P* < 0.01, ****P* < 0.001, *****P* < 0.0001 by one-way ANOVA followed by Dunnett's multiple comparisons test. (H) Schematic model of the three different isoforms of LINC01578. (I) The expression of the three different isoforms of LINC01578 in colon cancer cells was measured by qRT-PCR with isoforms-specific primers. Data are shown as mean ± SD based on three independent experiments. **P* < 0.05, ***P* < 0.01, ****P* < 0.001, ns, not significant, by one-way ANOVA followed by Tukey's multiple comparisons test. (J) The expression of the three different isoforms of LINC01578 in colon cancer tissues was measured by qRT-PCR with isoforms-specific primers. Data are shown as mean ± SD based on three random tissues. ***P* < 0.01, ns, not significant, by one-way ANOVA followed by Tukey's multiple comparisons test. (K) The levels of LINC01578 in purified polyadenylated RNAs. GAPDH and U6 served as poly (A) positive and negative control, respectively. Data are shown as mean ± SD based on three independent experiments.

**Fig. S2.** NF-κB and YY1 activated LINC01578 expression in HT-29 cells. (A) LINC01578 expression in HT-29 cells treated with PBS or 10ng/ml TNF-α for 24 hours. (B) LINC01578 expression in HT-29 cells treated with DMSO or 5µM JSH-23 for 24 hours. (C) LINC01578 expression in HT-29 cells transfected with p65 overexpression vector. (D) LINC01578 expression in HT-29 cells transfected with siRNAs against p65. (E) LINC01578 expression in HT-29 cells transfected with YY1 overexpression vector. (F) LINC01578 expression in HT-29 cells transfected with siRNAs against YY1. Data are shown as mean ± SD based on three independent experiments. ***P* < 0.01, ****P* < 0.001 by Student’s *t*-test.

**Fig. S3.** Overexpression of LINC01578 enhanced DLD-1 cell viability and mobility. (A) LINC01578 expression in another DLD-1 clone with stable LINC01578 overexpression. (B) Cell viability of LINC01578 overexpressed and control DLD-1 cells was determined by Glo cell viability assay. (C) Cell proliferation of LINC01578 overexpressed and control DLD-1 cells was determined by EdU assays. Scale bars, 100 µm. (D) Cell migration of LINC01578 overexpressed and control DLD-1 cells was determined by transwell migration assays. Scale bars, 100 µm. (E) Cell invasion of LINC01578 overexpressed and control DLD-1 cells was determined by transwell invasion assays. Scale bars, 100 µm. Data are shown as mean ± SD based on three independent experiments. ***P* < 0.01, ****P* < 0.001 by Student’s *t*-test.

**Fig. S4.** Depletion of LINC01578 repressed HT-29 cell viability and mobility. (A) LINC01578 expression in HT-29 cells infected with shRNAs targeted to LINC01578. (B) Cell viability of LINC01578 depleted and control HT-29 cells was determined by Glo cell viability assay. (C) Cell proliferation of LINC01578 depleted and control HT-29 cells was determined by EdU assays. Scale bars, 100 µm. (D) Cell migration of LINC01578 depleted and control HT-29 cells was determined by transwell migration assays. Scale bars, 100 µm. (G) Cell invasion of LINC01578 depleted and control HT-29 cells was determined by transwell invasion assays. Scale bars, 100 µm. Data are shown as mean ± SD based on three independent experiments. ***P* < 0.01, ****P* < 0.001 by one-way ANOVA followed by Dunnett's multiple comparisons test.

**Fig. S5.** LINC01578 repressed IκBβ and activated YY1. (A) p50 and p65 activation in nuclear extracts from LINC01578 overexpressed and control DLD-1 cells treated with DMSO or 5µM JSH-23 was determined by NFκB p50 Transcription Factor Assay Kit and NFκB p65 Transcription Factor Assay Kit, respectively. (B) p50 and p65 activation in nuclear extracts from LINC01578 overexpressed and control DLD-1 cells treated with DMSO or 5µM Bay 11-7085 was determined by NFκB p50 Transcription Factor Assay Kit and NFκB p65 Transcription Factor Assay Kit, respectively. (C) IκBβ expression in liver metastatic tumors derived from LINC01578 overexpressed and control DLD-1 cells was determined by qRT-PCR and western blot. (D) IκBβ expression in liver metastatic tumors derived from LINC01578 depleted and control LoVo cells was determined by qRT-PCR. (E) The in vitro transcribed LINC01578 and antisense LINC01578 used for RNA pull-down assays were detected by RNA electrophoresis. (F) YY1 IHC staining of liver metastatic tumors derived from LINC01578 overexpressed and control DLD-1 cells. Scale bars, 50 µm. (G) YY1 IHC staining of liver metastatic tumors derived from LINC01578 depleted and control LoVo cells. Scale bars, 50 µm. Data are shown as mean ± SD based on three independent experiments (A,B) or n = 6 mice in each group (C,D,F,G). **P* < 0.05, ***P* < 0.01, ns, not significant, by Student’s *t*-test (A,B), Mann-Whitney test (C,F), or Kruskal-Wallis test followed by Dunn's multiple comparisons test (D,G).
